# EU surveys insights: analytical tools, future directions, and the essential requirement for reference materials in wastewater monitoring of SARS-CoV-2, antimicrobial resistance and beyond

**DOI:** 10.1186/s40246-024-00641-5

**Published:** 2024-06-27

**Authors:** Valentina Paracchini, Mauro Petrillo, Anandasagari Arcot Rajashekar, Piotr Robuch, Ursula Vincent, Philippe Corbisier, Simona Tavazzi, Barbara Raffael, Elisabetta Suffredini, Giuseppina La Rosa, Bernd Manfred Gawlik, Antonio Marchini

**Affiliations:** 1 European Commission, Joint Research Centre (JRC), Geel, Belgium; 2 Seidor Italy SRL, Milano, Italy; 3 European Commission, Joint Research Centre (JRC), Ispra, Italy; 4 Department of Food Safety, Nutrition and Veterinary Public Health, Istituto Superiore di Sanità (ISS), Rome, Italy; 5 National Center for Water Safety (CeNSia), Istituto Superiore di Sanità (ISS), Rome, Italy

**Keywords:** Public health, Wastewater surveillance, SARS-CoV-2, AMR, Analytical workflow, Standards, Reference materials

## Abstract

**Background:**

Wastewater surveillance (WWS) acts as a vigilant sentinel system for communities, analysing sewage to protect public health by detecting outbreaks and monitoring trends in pathogens and contaminants. To achieve a thorough comprehension of present and upcoming practices and to identify challenges and opportunities for standardisation and improvement in WWS methodologies, two EU surveys were conducted targeting over 750 WWS laboratories across Europe and other regions. The first survey explored a diverse range of activities currently undertaken or planned by laboratories. The second survey specifically targeted methods and quality controls utilised for SARS-CoV-2 surveillance.

**Results:**

The findings of the two surveys provide a comprehensive insight into the procedures and methodologies applied in WWS. In Europe, WWS primarily focuses on SARS-CoV-2 with 99% of the survey participants dedicated to this virus. However, the responses highlighted a lack of standardisation in the methodologies employed for monitoring SARS-CoV-2. The surveillance of other pathogens, including antimicrobial resistance, is currently fragmented and conducted by only a limited number of laboratories. Notably, these activities are anticipated to expand in the future. Survey replies emphasise the collective recognition of the need to enhance the accuracy of results in WWS practices, reflecting a shared commitment to advancing precision and effectiveness in WWS methodologies.

**Conclusions:**

These surveys identified a lack of standardised common procedures in WWS practices and the need for quality standards and reference materials to enhance the accuracy and reliability of WWS methods in the future. In addition, it is important to broaden surveillance efforts beyond SARS-CoV-2 to include other emerging pathogens and antimicrobial resistance to ensure a comprehensive approach to protecting public health.

**Supplementary Information:**

The online version contains supplementary material available at 10.1186/s40246-024-00641-5.

## Background

Wastewater surveillance (WWS), based on the analysis of sewage, offers a unique approach to gather information on human populations at a community level [1, 2]. Wastewater systems serve as interconnected networks linking households, hospitals, animal farms, and agriculture, providing a conduit for microorganisms and their resistance [3].

Urban sewage, being a source of sampling material from a large community including healthy as well as asymptomatic and symptomatic individuals, overcomes ethical and logistical challenges associated with direct population sampling [4]. Its effectiveness has been demonstrated in global initiatives like the successful monitoring of poliovirus, severe acute respiratory syndrome coronavirus 2 (SARS-CoV-2) and many other pathogens [5–8]. This rapid and cost-effective approach offers valuable insights into the presence and prevalence of pathogens (viruses, bacteria, parasites, fungi), and antimicrobial resistance (AMR) genes at a community level [2, 6, 9, 10]. Studies have shown a high correlation between wastewater data and clinical data and therefore WWS has been proposed as a valuable and independent approach complementing traditional clinical epidemiology [11–14]. WWS effectively monitors the ongoing circulation of pathogens within a population and can function as an early warning tool by providing near real-time evidence before clinical reports are available [15–22]. This powerful approach has the potential to provide public health officials with early warning signals and crucial information for informed decision-making, enabling them to (i) implement targeted interventions to mitigate outbreaks, (ii) allocate resources effectively to areas with the highest risk and (iii) monitor the effectiveness of control measures. The European Commission proposal for a revised Urban Waste Water Treatment Directive underscores the significance of sewage surveillance for early warning purposes, recognising sewage treatment plants as potential transmission pathways for pathogens and WWS as a way to monitor the spreading of a certain pathogen [23]. In response to the COVID-19 pandemic in 2021, the European Commission recommended wastewater monitoring across EU Member States (Commission Recommendation (EU) 2021/472) [24]. Over a thousand wastewater treatment plants across the EU have been consistently monitored, enhancing coordinated decision and facilitating early detection of the virus [25, 26]. Looking ahead, the proven effectiveness of WWS in the context of COVID-19 paves the way for broader surveillance of emerging pathogens, drugs, pharmaceuticals, pollutants and AMR [27]. The specification of targets and parameters, including monitoring frequency and specific locations, continues to be the subject of active discussions among the various stakeholders and the scientific community. This dynamic process may need to adapt to new emerging health threats [23, 24].

AMR is recognised as a global threat to human, animal, and ecosystem health [28]. AMR surveillance is crucial for understanding trends, monitoring interventions, and designing guidelines, as outlined in the WHO AMR action plan [29, 30]. Given the complexity of the AMR problem, it requires specific actions in the fields of human health, animal health and the environment through the ‘One Health’ approach [31] as also recommended by the United Nations Environment Programme [32] and the European Council [33, 34]. Within this framework, the role of WWS in AMR surveillance has been highlighted [35, 36].

Nevertheless, the use of varied analytical methods, protocols, and quality controls in WWS presents challenges in guaranteeing comparability across results obtained from different laboratories [37]. Reference materials play a crucial role in harmonising and standardising results, offering a consistent benchmark for accuracy and precision across different laboratories, instruments, and methods. However, reference materials specifically designed for WWS, whether certified or not, remain scarce, often replaced by homemade alternatives, tailored for specific locations or laboratories. The development and use of reference materials would improve the reliability and comparability of results across different laboratories.

In response to the evolving situation of WWS and the need to systematically review current and anticipated practices, particularly the methodologies and workflows used by laboratories and the necessity for reference materials, two surveys were launched in July 2023 within the European Union and beyond. Survey 1, titled “Reference Materials in Wastewater Surveillance”, focused on the current practices and post-COVID-19 initiatives of WWS laboratories in monitoring various pathogens and AMR in wastewater. The main goals of this survey were to provide a snapshot of existing practices, identify the pathogens monitored, understand gaps in the field, address challenges faced by practitioners, and pinpoint potential areas for improvement.

Survey 2, titled “Reference Materials in Wastewater Surveillance for SARS-CoV-2”, specifically examined the surveillance of SARS-CoV-2 in wastewater. It investigated the methodological procedures and quality controls developed and implemented by laboratories for the detection, quantification, and monitoring of SARS-CoV-2. The primary objective was to determine the need for procedure standardisation and the development of reference materials to improve the quality and comparability of results.

This manuscript aims to present and discuss the results from these surveys, describing the current practices and identifying challenges and opportunities for standardisation and improvement in wastewater surveillance methodologies. We anticipate that these results will assist the various actors operating in the WWS arena to pinpoint critical areas for improvements and in setting future priorities including the selection of key reference materials to improve the quality of the results and harmonisation. This will further strengthen the pivotal role of WWS within the EU and beyond.

## Methods

### Selection of participants

The surveys aimed to gather information on wastewater practices in Europe, specifically focusing on national laboratories within the EU Wastewater Observatory for Public Health Network (https://wastewater-observatory.jrc.ec.europa.eu/). This network results from the international activities linked to implementing and institutionalising wastewater-based surveillance for public health, including the animation of an active and regular Engagement Mechanism with the Community of Practices. Invitations to participate were sent via email to available addresses within the Network, resulting in a total of 797 e-mails sent. During the time the survey was open, we solicited invited laboratories by sending two kind reminders.

### Design of the surveys

The two surveys were designed and launched on 12 July 2023. Survey 1 included a total of nine questions in either multiple-choice or open-field format. The questions were crafted to evaluate the participants’ current WWS activities, their future plans, and to gauge their potential requirement for reference materials. The questionnaire is detailed in Additional File 1. The list of pathogens of the first question was selected considering pathogens for which wastewater and/or clinical surveillance had been already established. In addition, the survey included the option “Other” where participants could eventually specify any other pathogen not included in the list.

In parallel with the Survey 1, Survey 2 was also launched. The survey comprised two primary sections. The first part included ten questions designed to gain a broad understanding of the workflow and technical protocols adopted by laboratories for detecting, quantifying, and monitoring the spread of SARS-CoV-2 in wastewater samples. The second part, consisting of nine questions, focused on gathering information from participants regarding the specific quality controls implemented in their procedures and the need for reference materials. The questionnaire is available as Additional File 2.

The two surveys remained open on the EU survey platform (https://ec.europa.eu/eusurvey/) until 10 September 2023.

### Analysis of the results

Microsoft Excel and PowerBI were used for data analysis and graphical representation of results. Results were initially screened to eliminate eventual duplication in replies and responses from the same laboratory were collected, merged and treated as a single reply.

Antimicrobial genes were assigned to their respective classes using the official nomenclature provided by NCBI (https://www.ncbi.nlm.nih.gov/pathogens/antimicrobial-resistance/).

## Results

### EU survey participation

Out of the 797 invitations sent to participate in the surveys, 671 (84%) were distributed within Europe. Table 1 details the distribution of invitations by continent, along with the number of responses received for each of the two surveys. It is important to note that while the survey was open to regions beyond Europe, the majority of responses received were from European laboratories. This provides a comprehensive picture of WWS in Europe, contrasting with the fragmented view gathered from other parts of the world.

**Table 1:** Number of invitations sent per continent and number of replies obtained

Continent	Invitations	Number of replies for Survey 1	Number of replies for Survey 2
Africa	13	0	0
Asia	26	5	1
Europe	671	101	70
North America	68	12	10
Oceania	19	1	1
**TOTAL**	**797**	**119**	**82**

A total of 139 replies were received for the Survey 1. However, four responses were identified and excluded due to duplications, while 29 responses were merged into 13 due to multiple submissions originating from the same laboratory. Therefore, a total of 119 replies were considered valid for analysis (Table 1). Of these, 101 replies were from Europe, accounting for 85% of the total. Among European respondents, 50% were from National Laboratories, ensuring broad coverage across a majority of European countries. The remaining responses included 20% from industry / private sector and 30% from academia.

**Fig. 1:**
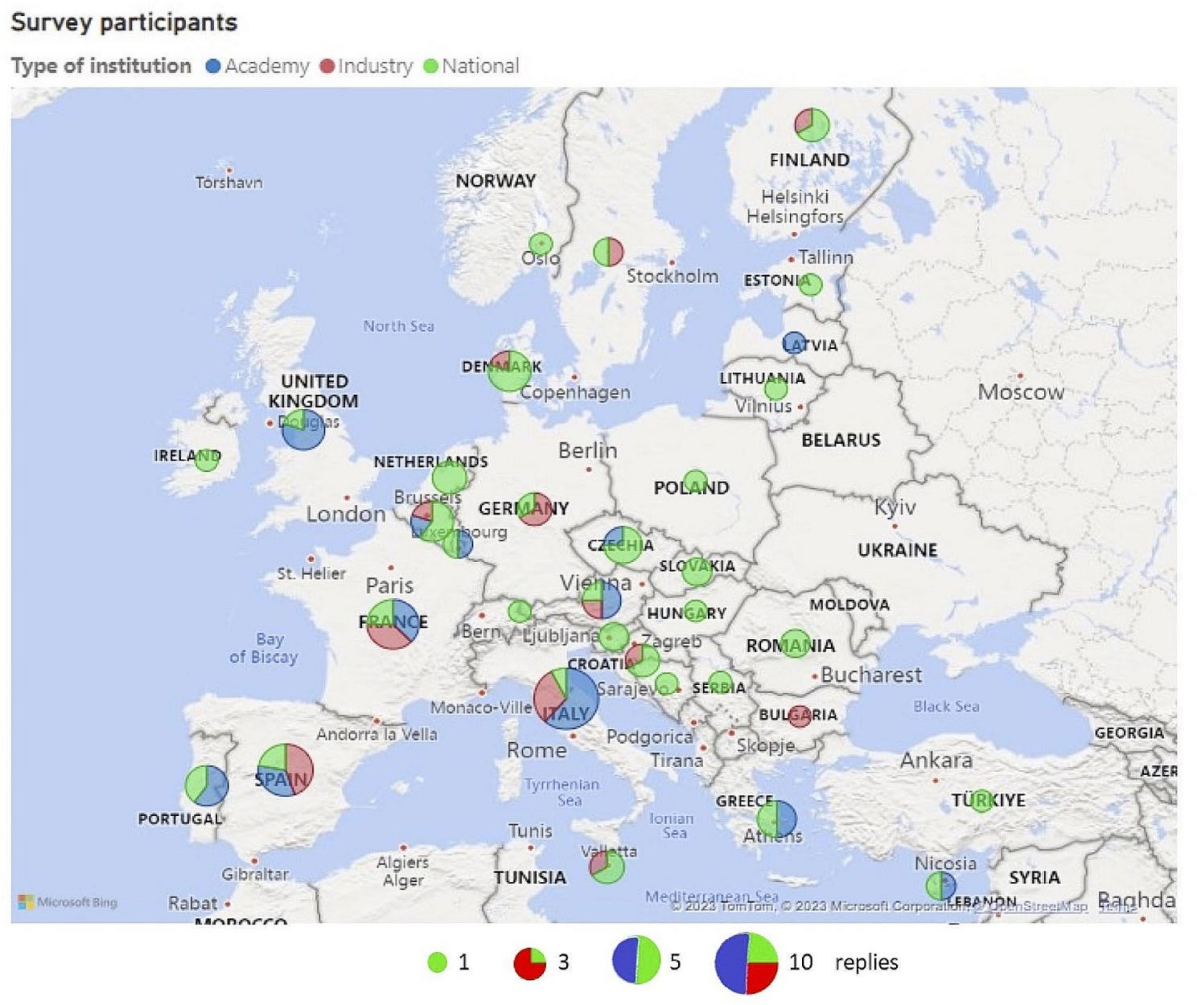
Replies to the Survey 1 by country within Europe. Replies are illustrated with bubbles, where the bubble size correlates with the number of participants

Figure 1 illustrates the distribution and number of responses to Survey 1, presented by country within Europe. For a detailed breakdown of participation by European countries, refer to Additional File 3. The worldwide distribution of replies is detailed in Additional File 4.

For the Survey 2, we received 85 replies. One response was excluded due to duplication, and four responses from the same laboratories were consolidated into two. Consequently, 82 valid replies were considered for the analysis (Table 1). Of these, 43% were received from national laboratories, 20% from industry and 37% from academia.

## Survey 1

### Pathogens monitored in wastewater and environmental surveillance program

**Fig. 2:**
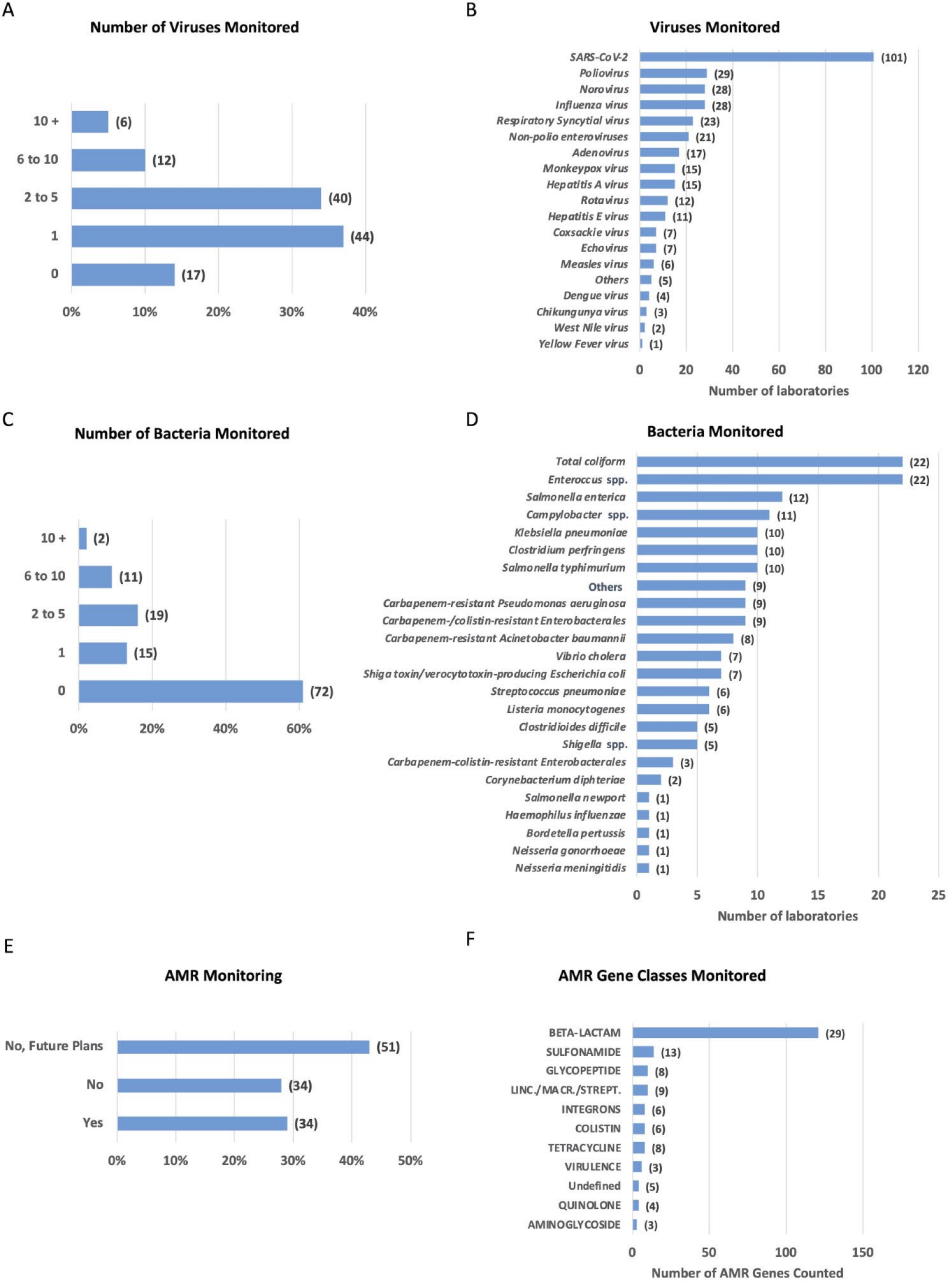
Viruses, bacteria, and AMR genes monitored in wastewater surveillance programs. **(A)** Variability in virus monitoring across laboratories. Columns indicate the percentage of laboratories monitoring varying numbers of viruses, with numbers in brackets indicating the corresponding laboratory counts for each category. **(B)** Monitored viruses. Columns depict the number of laboratories monitoring the indicated viruses. **(C)** Variability in bacteria monitoring across laboratories. Columns indicate the percentage of laboratories monitoring varying numbers of bacteria, with numbers in brackets indicating the corresponding laboratory counts for each category. **(D)** Bacteria monitored. Columns depict the number of laboratories monitoring the indicated bacteria. **(E)** Percentage of laboratories monitoring AMR genes. Columns indicate the fraction of laboratories performing or not the monitoring of AMR genes and those that are planning to do it in the future. Numbers in brackets represent the total number of the laboratories. **(F)** AMR genes monitoring by class. Columns indicate the number of monitored AMR genes grouped by indicated classes. Numbers in brackets represent the number of laboratories testing AMR genes within each specified class

In terms of virus monitoring, 102 out of 119 laboratories (86% of the total) specified that they do test viruses (see Additional File 5 A for information about the type of laboratories involved in the monitoring). Among them, 44 laboratories (37%) test only one virus, 40 laboratories (34%) test between 2 and 5 viruses, 12 (10%) test between 6 and 10 viruses, and 6 (5%) test more than 10 viruses (Fig. 2A). Among the 102 laboratories testing viruses, all but one monitor SARS-CoV-2. The other most commonly monitored viruses are Poliovirus, Influenza and Norovirus, followed by Respiratory Syncytial virus and non-polio Enteroviruses. Four laboratories monitor emerging, rodent-borne, and zoonotic viral pathogens (such as Dengue, West Nile, Chikungunya, Yellow Fever viruses) (Fig. 2B). Only 17 laboratories (14%) do no test any virus (Fig. 2A).

In terms of bacteria monitoring, 47 out of 119 laboratories (39%) specified that they monitor bacteria. Among these, 15 laboratories test only one bacterium in their routine analyses, while only 2 laboratories test more than 10 different bacteria (Fig. 2C and Additional File 5B). The bacteria most commonly monitored by these laboratories include *Enterococcus* spp and total *coliform* bacteria, followed by *Salmonella enterica* and *Salmonella typhimurium*, *Campylobacter* spp., *Klebsiella pneumoniae* and *Clostridium perfringens*. Regarding antibiotic resistant bacteria, the most frequently monitored are Carbapenem-resistant *Pseudomonas aeruginosa*, Carbapenem-/colistin-resistant *Enterobacterales* and Carbapenem-resistant *Acinetobacter baumannii* (Fig. 2D).

Out of 119 laboratories, only 34 acknowledged analysing the presence of AMR genes in their surveillance programs, another 34 do not monitor AMR genes, while 51 of them plan to do so in the future (Fig. 2E and Additional File 5 C). Among the monitored AMR genes, the majority belong to the Beta-Lactam class (including subclasses Carbapenem, Cephalosporin, and Beta-Lactam) (Fig. 2F).

**Fig. 3:**
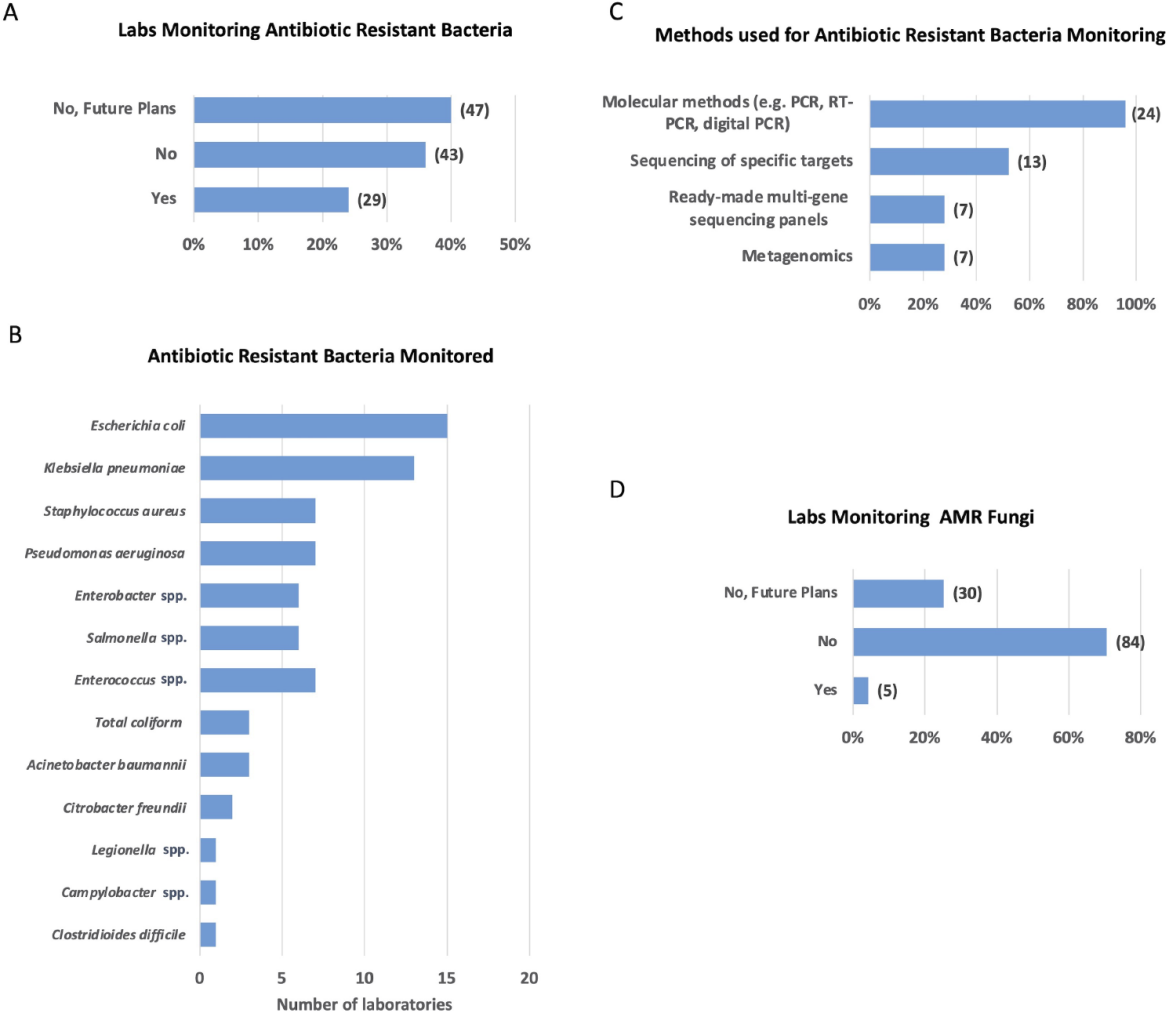
Laboratories involved and methods used for the monitoring of antibiotic resistant bacteria and AMR fungi. **A.** Percentage of laboratories monitoring antibiotic resistant bacteria. The graph indicates the fraction of laboratories currently performing or not the analyses and those that are planning to do it in the future. Numbers in brackets represent the total number of the laboratories. **B.** Monitored antibiotic resistant bacteria. Bars show the number of laboratories monitoring the indicated bacteria. **C.** Methods for monitoring antibiotic-resistant bacteria. Bars illustrate the fraction of laboratories employing indicated methods for surveillance, with numbers in brackets indicating the corresponding count of laboratories utilising each method. **D**. Percentage of laboratories monitoring AMR fungi. Bars indicate the percentage of laboratories currently monitoring or not fungi bearing AMR and those that are planning to initiate monitoring in the future. Numbers in brackets represent the number of laboratories

Twenty-nine laboratories reported to monitor antibiotic-resistant bacteria, while the rest either does not monitor (43 laboratories) or plan to do so in the future (47 laboratories) (Fig. 3A and Additional File 5D). Among the most monitored antibiotic-resistant bacteria are resistant *Escherichia coli*, *Klebsiella pneumoniae, Staphylococcus aureus* and *Pseudomonas aeruginosa* (Fig. 3B). Molecular methods, including quantitative PCR, Real Time Reverse Transcriptase (RT) PCR, and digital PCR, are predominantly employed for monitoring these bacteria (24 laboratories), often complemented by sequencing of specific targets (13 laboratories), metagenomics (7 laboratories), and the utilisation of ready-made multi-gene sequencing panels (7 laboratories) (Fig. 3C).

When it comes to fungi harbouring AMR, only 5 laboratories reported measuring their presence, while 30 laboratories expressed their intention to conduct this monitoring in the future (Fig. 3D and Additional File 5E). The monitored fungi include *Candida albicans* and *Aspergillus fumigatus.*

The term ‘environmental surveillance’ extends beyond wastewater surveillance, including monitoring in freshwaters, air, soil, food, and other surfaces. Out of 119 laboratories, 65 reported conducting environmental surveillance. These surveillance programs primarily focus on monitoring bacteria (mostly *Enterococci*) and viruses (mainly SARS-CoV-2) in freshwaters including roof water used for recreational purposes, soil, and in various materials such as sludge, stool, sediment, manure, biofilm and food. Additionally, a few laboratories indicated testing for other organisms, such as fungi (3 laboratories) and parasites (5 laboratories). Furthermore, two laboratories specified that they perform chemical analyses to assess environmental surveillance (Additional File 5 F and Additional File 6).

### Need to improve the accuracy of the results

To assess the necessity for implementing quality controls or reference materials in WWS, we requested the laboratories to provide feedback on the need to improve the accuracy of their results.

Ninety-three laboratories expressed the need to improve results accuracy, while 17 reported no need for improvement, with 9 not responding to that question.

**Fig. 4:**
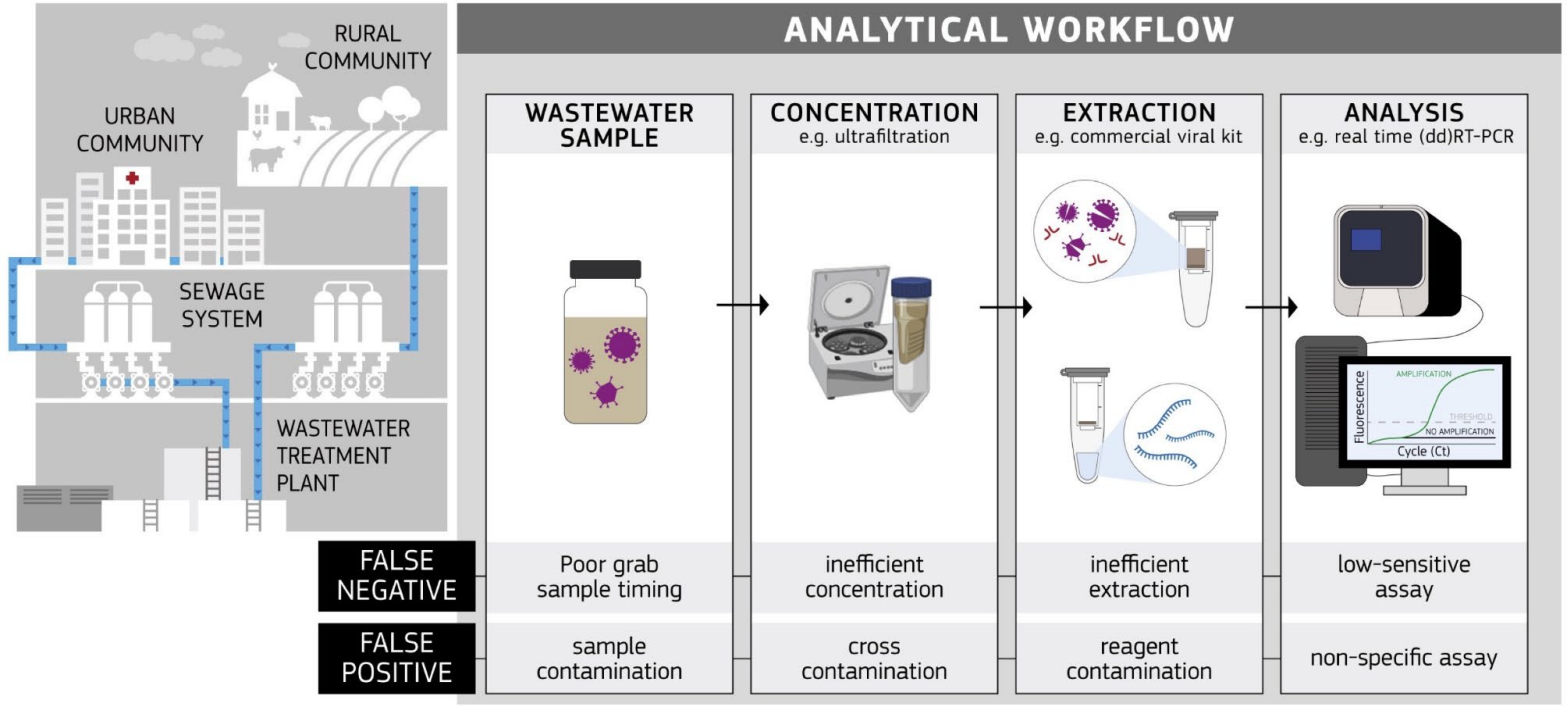
Schematic representation of the analytical workflow from sample collection to the analysis of SARS-CoV-2 (or other pathogens). Wastewater samples are collected from influent or effluent points of wastewater treatment plants, strategically chosen to capture a representative snapshot of the served population. Sampling occurs at regular intervals to monitor temporal variations in pathogen concentrations. Potential errors, such as false negatives or false positives, can occur at each processing step, with main sources of errors indicated

In WWS, reference materials play a key role in controlling and validating the analytical workflow (see Fig. 4), ensuring accurate results for detecting and quantifying pathogens or pollutants. These materials, ranging from whole organisms like heat-inactivated viruses to nucleic acid standards, may be used as internal controls to verify, for instance, the effectiveness of sample concentration, nucleic acid extraction, and/or the accuracy and reliability of downstream analytical techniques such as RT-PCR-based assays and sequencing.

**Fig. 5:**
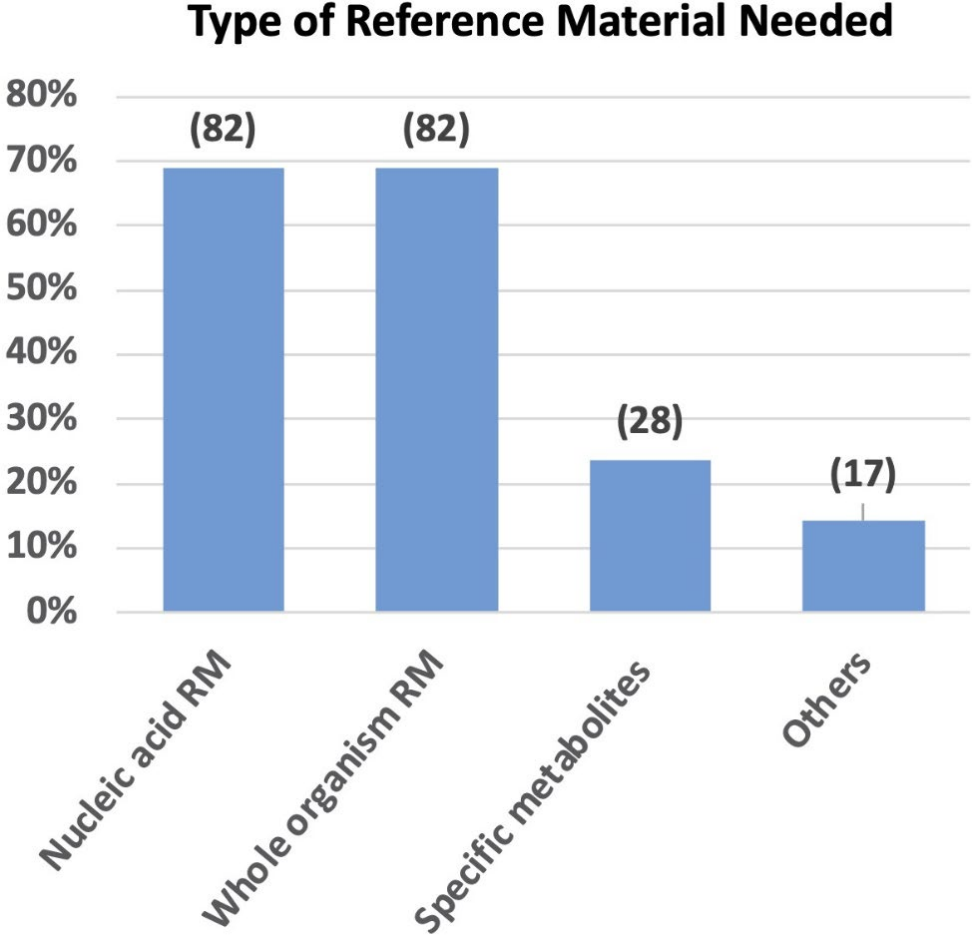
Preferences for reference materials. The graph illustrates the expression of interest for various classes of reference materials in WWS to improve the accuracy of the results. Numbers in brackets indicate the number of laboratories expressing preference for each category. Abbrev.: RM: reference materials

In terms of required reference materials, 82 laboratories expressed the need for both nucleic acid reference materials certified for their nucleotide sequence and target concentration, along with whole organism reference materials certified for their identity and concentration. Additionally, 28 laboratories highlighted the need for specific metabolites for chemical analyses at certified concentrations. Notably, 17 participants emphasised the necessity for other reference materials, such as clinical samples, spiked wastewater samples, or standardised wastewater containing different certified concentrations of metabolites/inhibitors combined with certified concentrations of relevant organisms/variants (Fig. 5).

## Survey 2

### WWS surveillance of SARS-CoV-2

The Survey 2 aimed at collecting information on different workflows and technical protocols adopted by laboratories for detecting, quantifying, and monitoring SARS-CoV-2 in wastewater samples.

The analysis of SARS-CoV-2 (and other pathogens) in wastewater involves a well-defined workflow that includes sample collection, processing, and laboratory analysis. Figure 4 provides an overview and a step-by-step description of the typical workflow for SARS-CoV-2 analysis in wastewater indicating major reasons for false negative and false positive results (Fig. 4).

**Fig. 6:**
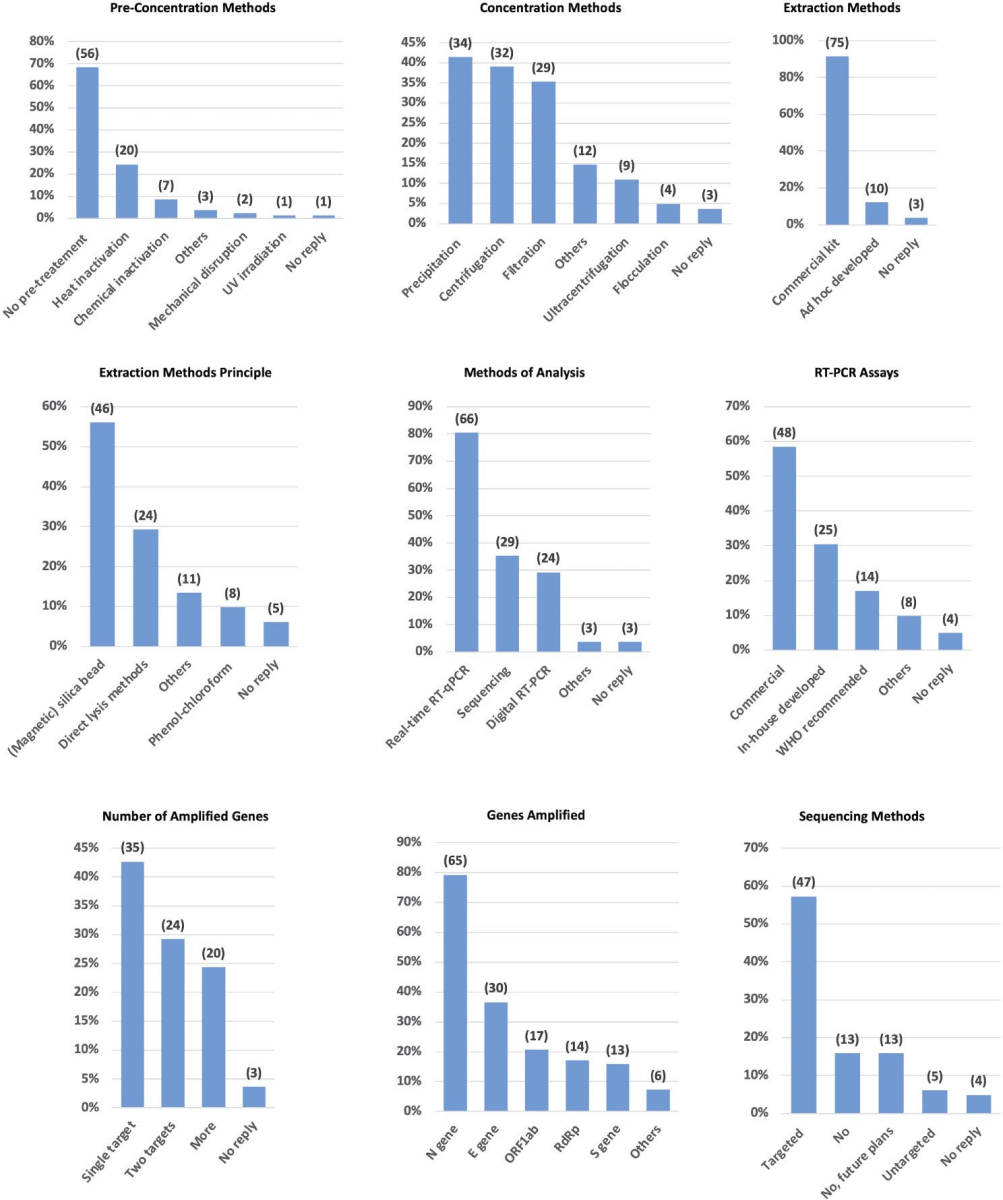
Overview of wastewater sample processing and testing for SARS-CoV-2. The figure illustrates the methods used by WWS laboratories for the sample pre-concentration, concentration, RNA extraction and RNA analysis. It includes details on RNA extraction principles, RT-PCR assays employed, number and type of genes amplified, and sequencing methods. Columns show the relative percentages in relation to total responses, with numbers on top indicating the total number of laboratories using each specific method

The workflow often starts with a pre-concentration step aiming at virus inactivation. The results of the survey indicate that the majority of the laboratories (56 out of 82) do not perform any pre-treatment of the sample to inactivate the virus. Among the treatments and agents that can be used to inactivate RNA viruses, heat inactivation is the prevalent choice (carried out by 20 laboratories) followed by chemical inactivation (7 laboratories) (Fig. 6).

Filtration, centrifugation, and other concentration techniques are employed to concentrate viral particles from the bulk of the wastewater. The survey results show that precipitation using polyethylene glycol (PEG) or organic solvents is commonly used (34 laboratories), followed by centrifugation (32 laboratories) and filtration (29 laboratories). Additionally, nine laboratories use ultracentrifugation and four flocculation (Fig. 6).

The concentrated samples are subject to RNA extraction procedures to isolate the virus’s genetic material. Commercial RNA extraction kits or ad-hoc developed methods are utilised by 75 and 10 laboratories, respectively (Fig. 6). These extraction methods are based on different principles, with the majority of the laboratories using magnetic silica bead-based extraction methods (46 laboratories), followed by direct lysis methods (24 laboratories) (Fig. 6).

Different methods of analysis are applied for the detection of SARS-CoV-2: RT-PCR (66 laboratories), sequencing (29 laboratories) or digital RT-PCR (24 laboratories) are commonly used by the participants to the survey (Fig. 6).

Laboratories employ various RT-PCR assays with a predominant use of commercial assays (48 laboratories), followed by in-house developed assays (25 laboratories), and WHO recommended assays (14 laboratories). These assays are designed to amplify one or more SARS-CoV-2 targets. Out of the 82 participants, 35 amplify a single target gene, 24 analyse two targets and 20 more than two targets. From the results, considering all the assays together (in simplex, duplex, or multiplexing), the N gene is the target preferentially amplified, followed by the E gene, the ORF1ab and RdRp gene (Fig. 6).

Among the 52 laboratories performing sequencing for the analysis of the genome of SARS-CoV-2 variants, 47 use a targeted approach based on amplicon sequencing, with only five opting for a metagenomics approach. The remaining laboratories do not currently use sequencing methods, but 13 of them expressed the intention to use them in the future (Fig. 6).

Similar results were obtained when restricting the analysis exclusively to the national laboratories (Additional File 7).

### Quality controls adopted in SARS-CoV-2 WWS

**Fig. 7:**
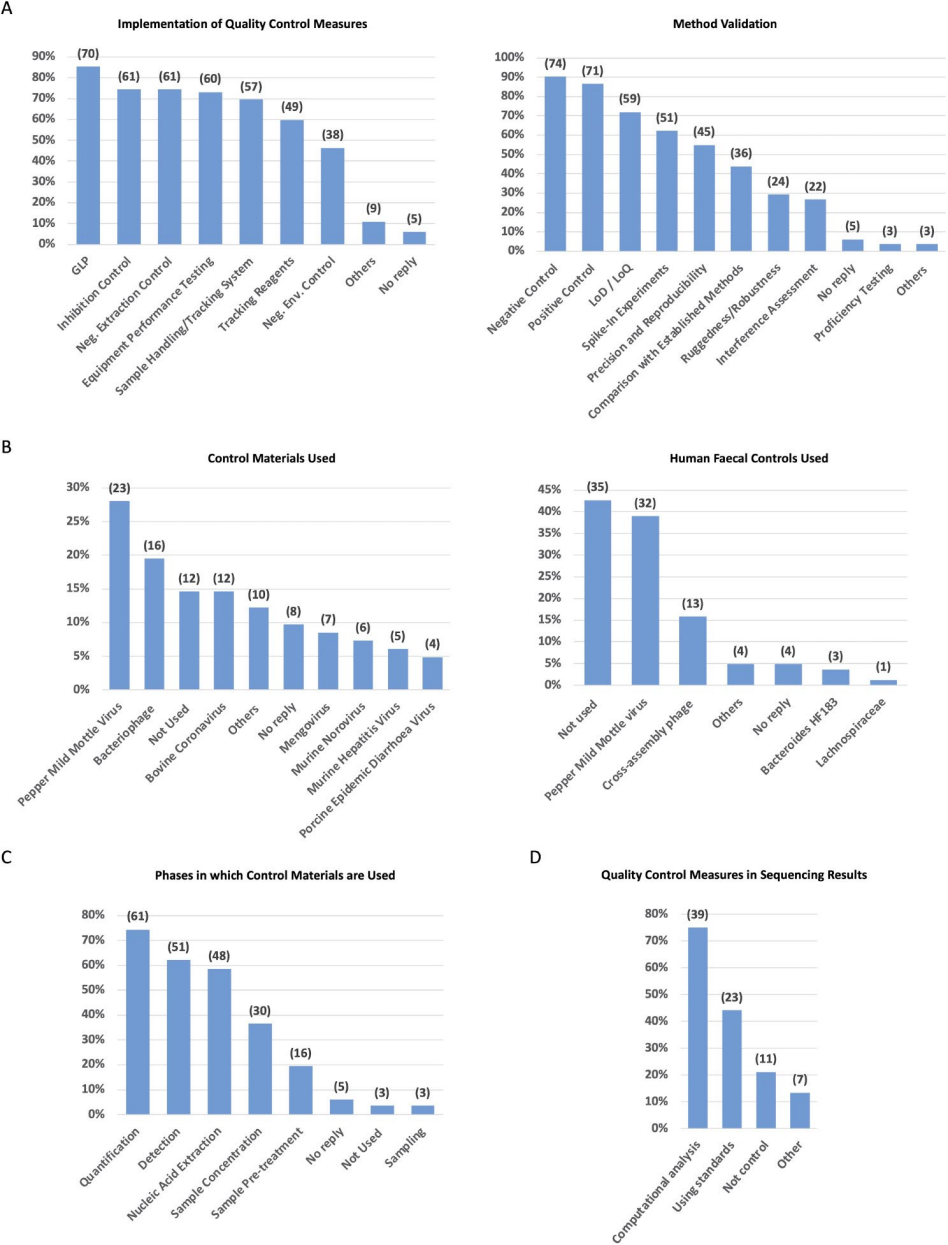
Quality control measures applied in SARS-CoV-2 wastewater surveillance **(A)** Quality assurance and validation procedures in laboratories. Columns depict the fraction of laboratories implementing the diverse quality control measures in current practices (left panel) and during method validation (right panel), with numbers on top indicating the total number of laboratories. **(B)** Reference materials. Columns represent the fraction of laboratories using the diverse internal controls (left panel) and faecal content controls (right panel), with numbers indicating the total number of laboratories using that specific material. **(C)** Utilisation of control materials across workflow phases. Columns represent the fraction of laboratories using control materials during the various phases of their workflow, with numbers on top indicating the count of laboratories using the material in each specific phase. **(D)** Quality control measures for sequencing results. Columns represent the percentage of laboratories implementing different control measures to ensure the quality of sequencing results. Numbers on top represent the number of laboratories employing each specific measure. Abbrev.: GLP: good laboratory practice; LoD: limit of detection; LoQ: limit of quantification; Neg.: negative

Most of laboratories have implemented a comprehensive set of quality control measures to ensure the effective execution of their workflows. These measures encompass adherence to good laboratory practices, meticulous monitoring and documentation of equipment performance, and the maintenance of rigorous sample handling and tracking systems. Additionally, laboratories often control presence of PCR inhibitors (inhibition control) and use negative environmental controls (Fig. 7A left panel).

The replies reveal that a majority of laboratories integrate negative (74/82) and positive (71/82) controls in their analyses and method validation (Fig. 7A right panel). Approximately half of these laboratories also assess the limit of quantification (LoQ) and limit of detection (LoD), as well as the precision of their methods and reproducibility of their results. A smaller percentage takes the additional step of validating methods through spike-in experiments and comparing them with other established standardised methods (Fig. 7A right panel). This tendency may be attributed to the widespread use of commercial kits and a reliance on manufacturers’ declared performance. Notably, only a few laboratories have participated in proficiency testing (PT) exercises, benchmarking their capabilities against peers (Fig. 7A right panel).

An overwhelming majority of laboratories (72/89) acknowledged the use of (internal) control materials in their workflow to assess virus recovery rate. The diverse selection includes various members of the Coronavirus family, such as bovine coronavirus, porcine epidemic diarrhoea virus, and mouse hepatitis coronavirus, along with surrogate viruses like murine norovirus, bacteriophages, and pepper mild mottle virus, reflecting the heterogeneous approaches in ensuring accuracy, precision, and reliability in laboratory analyses (Fig. 7B left panel).

Wastewater, as a matrix, undergoes significant fluctuations in terms of composition influenced by factors such as precipitation patterns, pH, population density, and seasonal changes. Survey 2 highlighted that most of the responding laboratories do not use human faecal control for normalising their results based on faecal content. Among the laboratories using faecal controls, the most used is the pepper mild mottle virus (Fig. 7B right panel).

Control materials are essentially used at the final steps of the workflow and in particular during the nucleic acid extraction and virus detection or quantification (Fig. 7C).

Laboratories using sequencing technologies (52/82) were asked about the control measures implemented to ensure the quality of their results. A lack of consistency emerged, with a majority relying on computational analysis (39/52) (Fig. 7D). Twenty-three laboratories reported to validate the bioinformatics workflow using either international or national standards.

In conclusion, the survey underscores the diversity of approaches used by participating laboratories, emphasising the need for consensus on common procedures and/or the use of reference materials to ensure data alignment and comparability of results.

## Discussion

Urban wastewater is a direct by-product of human activities in urban environments, with its composition reflecting the occurrence and levels of microbiological, chemical, and physical pollutants in the population. In recent years, growing evidence supports the use of WWS as an indicator for the presence and detection of circulating pathogens within the community [2, 38, 39]. Furthermore, urban wastewater serves as a reservoir and source for AMR, making monitoring AMR in wastewater a crucial tool to comprehend the burden, transmission, and persistence of AMR in society [40, 41].

The dual purpose of the two surveys was to assess the current state of WWS practices mainly in Europe and to pinpoint gaps and limitations in existing methodologies and practices. Additionally, the surveys aimed to evaluate the specific needs of laboratories to address these gaps, and to anticipate future challenges within the WWS community.

The primary focus of WWS in Europe is on monitoring SARS-CoV-2, with nearly all participating laboratories engaged in this task. This focus is unsurprising and reflects the collective efforts and commitment directed towards efficient SARS-CoV-2 surveillance aiming to monitor the spread of the virus and mitigate its adverse effects throughout the pandemic.

During the COVID-19 pandemic, the use of WWS for SARS-CoV-2 and its variants has emerged as a powerful independent and complementary tool to clinical epidemiology, providing valuable insights into the virus’s presence and circulation within a certain community in a timely manner [42]. Several laboratories have shown that the detection of new SARS-CoV-2 variants in wastewater samples precedes its identification at the clinical level [19, 42, 43]. This emphasises the role of WWS as a proactive and early warning system, offering valuable insights for both the ongoing monitoring of SARS-CoV-2 and the timely identification of new viral variants within the population [16]. However, the potential of WWS for monitoring trends of current and future variants, while promising, is hindered by the complexity of differentiating between similar sublineages circulating at the same time. The analysis of the results deriving by high-throughput sequencing approaches requires sophisticated data analysis tools and specialised expertise and resources. In addition, sample quality and variability in wastewater composition can affect detection sensitivity and specificity, presenting challenges in tracking shifts in SARS-CoV-2 sequences across different regions and over time [44].

Numerous assays have been published in the scientific literature for the detection of SARS-CoV-2 in wastewater, and a number of papers evaluate the efficacy of different procedures applied in the analytical workflow [37, 45–50]. A recent review aiming at collecting information on different processes of wastewater sampling and testing, reported similar variability to what observed in our study (concentration techniques, RNA extraction, RT-PCR detection and target genes) and emphasised the need for constant efforts towards optimal strategies [51]. Additionally, the evolving nature of the virus poses a challenge as certain variants may elude detection by current assays [16]. Therefore, performance of the assays needs to be continuously verified and new assays eventually developed.

The surveys reveal that most of the participants have implemented an extensive arrays of quality control measures to guarantee the efficient execution of their workflows. This aligns well with the fact that many of the replies were received by national laboratories involved in SARS-CoV-2 surveillance programs. Most of these laboratories hold ISO accreditation (e.g. ISO/IEC 17025, ISO 15189), indicating the integration of general procedures into their workflows to maintain high-quality standards in their operations and services. This commitment to quality control ensures the reliability and accuracy of laboratory processes, contributing to the overall integrity of the generated results. Such commitment is further evidenced by participants’ declaration on method validation specific to their context.

However, monitoring SARS-CoV-2 in wastewater systems poses significant challenges [52, 53]. Despite extensive research efforts, a lack of standardised methods for viral RNA concentration, extraction, and quantification in WWS of SARS-CoV-2 complicates the comparison of data among laboratories. The absence of a uniform approach has led to variations in results, as observed in inter-laboratory comparisons conducted by various groups [52–54], with no single explanation accounting for such discrepancies. This lack of standardisation limits the effectiveness and reliability of WWS for SARS-CoV-2 monitoring [54]. The results of the surveys confirm this variability at large scale, considering not only academia and industry approaches but also the different methodologies used by national laboratories. The same outcome was also observed in recent literature reviews [55, 56] reporting heterogeneity and a lack of best practices concerning analytical procedures in SARS-CoV-2 WWS, reinforcing therefore the need for higher standards and quality controls to improve results accuracy, promote harmonisation and data comparability. Enhancing coordination, communication and fostering collaboration between (cross-border) laboratories may facilitate the exchange of experiences and the identification of best practices. The lack of standardised protocols and harmonised quality assurance and quality control (QA/QC) procedures, specifically underscores the necessity for certified reference materials in this domain. The development and utilisation of these certified reference materials is an important requirement towards establishing essential common benchmarks for ensuring precision, reliability, and consistency of results across various laboratories and studies.

While laboratories currently employ control materials, their use tends to be fragmented, heterogeneous, and limited to specific steps within the analytical workflow. Indeed, the survey results highlight the absence of a current consensus on the optimal WWS normalisation parameters e.g. to control the RNA extraction step and/or foecal content needed to establish even more robust correlations between SARS-CoV-2 WWS data and COVID-19 clinical case numbers. The use of surrogate viruses comes also with its weaknesses, as these viruses do not always resemble the physical and chemical characteristics of SARS-CoV-2 [57]. This reveals a critical gap and an urgent need for improvement, particularly in the development of reference materials certified for copy numbers concentration and sequence identity, ideally closely related to the target virus. The introduction of such materials, as spikes at the initial steps of the process, could significantly enhance the accuracy of pathogen recovery rate calculation. The use of faecal controls would also ensure accurate interpretation and comparison of data, accounting for variations in the composition of the matrix and providing more reliable insights into viral presence and trends.

It was also observed (with few exceptions) that participating laboratories did not engage in proficiency testing (PT) exercises for SARS-CoV-2 surveillance. The limited participation in PT exercises for SARS-CoV-2 surveillance may be attributed to the lack of organised PT initiatives specifically tailored for laboratories involved in WWS. This highlights the necessity of organising PTs to assess laboratory performance, identify potential gaps, uncover best practices, and foster the continual improvement of laboratory capability.

A recent review showed high variability in WWS sampling strategy around the world [42], while our study did not include it in its analysis, because it focussed on the WWS workflow after sampling. As highlighted by Tiwari et al. the selection of sampling strategy and relative frequencies, as well as the appropriate sample size and population coverage, should be defined according to pathogen epidemiological characteristics as, for example, stability over time [26].

The utility of WWS extends beyond COVID-19, as it has successfully aided in the monitoring and management of various infectious diseases, including Hepatitis A, Hepatitis E, Polio [2], Monkeypox [8], and campylobacteriosis [58]. In addition, wastewater serves as an important tool for the analysis of AMR patterns, providing valuable insights into the prevalence and evolution of antimicrobial-resistant microorganisms within communities [31, 35, 59].

Looking ahead, prioritising AMR monitoring becomes imperative, aligning with the WHO Global Action Plan on AMR. This includes monitoring drug resistance in fungi, an aspect not yet explored according to survey results. This initiative is closely intertwined with the overarching goal of reducing or optimising the use of antimicrobial medicines in both human and animal health. In line with these global efforts, the Council Recommendation, on stepping up EU actions to combat AMR, adopted in June 2023, sets ambitious targets for 2030 [34]. These targets include a 20% reduction in the total consumption of antibiotics in humans, illustrating the EU’s intensified efforts and commitment to combating AMR.

Recognised as one of the top 10 global public health threats by WHO [30, 60], AMR emerges prominently in the survey responses as a major concern for the future. However, the survey results reveal that AMR analysis in the wastewater community is still in its initial stages, involving less than 40% of the laboratories in this study and focuses mainly on beta-lactam resistant gram-negative bacteria, which are categorised as priority by WHO [59]. Our results confirm what was reported in a recent review [36], where it is stated that WWS on AMR has been more frequently evaluated for members of the *Enterobacteriaceae* family, mainly *Escherichia coli* and *Enterococcus* spp., and not tested consistently for all AMR pathogens. Similarly, a recent literature review compiling the latest development in AMR WWS in the Nordic European Countries [27] confirms our observations that pathogens producing Carbapenemase and extended-spectrum Beta-Lactamase were the most studied targets. In agreement with recent reports [35, 36, 40], the results of our surveys confirm knowledge gaps in AMR surveillance, as lack of standardised protocols and lack of standard monitoring targets and reference materials. Despite the advantages in offering a community-wide perspective, there is a clear need for improvements [40].

Addressing these deficiencies requires specific actions across various domains through a ‘One Health’ approach—a collaborative, trans-disciplinary, and multi-sectoral strategy promoting cooperation across human health, animal health, agrifood systems, and the environment as recently stated in the United Nations Environment Programme [32] and recommended by the European Council [33]. Researchers and public health professionals should work together to identify the microorganisms requiring control. This may involve the selection of common AMR markers and the establishment of acceptable concentration limits. These concerted efforts should provide clear guidance for effective intervention measures, ultimately mitigating the risks posed by specific pathogens to the community.

As observed for SARS-CoV-2, participants in our surveys express a critical need to enhance the accuracy of their results, with 80% acknowledging this imperative. A potential solution, identified by participants, is the use of reference materials, and the survey results indicate that whole organism reference materials or nucleic acid reference materials certified for their sequence and concentrations are the preferred choices among laboratories. In this respect, the whole organism reference material could be used as a spike material allowing to perform quality checks at different steps of the whole procedure, while the nucleic acid reference material could be used as a quality control for RT-PCR and sequencing. Harmonised results among laboratories analysing wastewater samples are crucial to align temporal trends, ensuring quantitative data expressed in a uniform unit. This standardisation is pivotal for establishing a comprehensive EU overview, especially in the face of emerging viruses, enabling effective monitoring and response across diverse laboratories and countries.

The insights gained from these surveys are expected to guide future directions in WWS, highlighting the importance of continued innovation and standardisation in the field. Moving forward in a post-COVID era, the lessons learned and the knowledge acquired will undoubtedly play a crucial role in shaping the development of resilient and robust public health surveillance systems, capable of detecting a wide range of pathogens and AMR in wastewater. High quality, comprehensive and real-time surveillance data are needed to support the use of WWS for regulatory purposes. This, in turn, will enhance our ability to respond to future public health challenges with greater speed and precision.

## Conclusions

- Survey results comprehensively identify the analytical procedures, monitored pathogens, future directions, challenges and gaps within the field of WWS, particularly at the European level.
- WWS in Europe primarily focuses on monitoring SARS-CoV-2.
- Despite collective efforts to enhance quality of results, laboratories employ diverse methods throughout the workflow for WWS of SARS-CoV-2. This multiplicity of approaches at various workflow steps underscores the ongoing challenge of harmonisation, revealing a lack of standardised common methods and reference materials.
- WWS of other pathogens, including AMR, is currently fragmented and confined to a small fraction of laboratories. However, there is an optimistic outlook, suggesting that this area is gaining attention from laboratories, and future efforts are anticipated to be more consistent and widespread.
- WWS of fungi bearing AMR is primarily addressed by a limited number of pioneering laboratories, with the majority yet to recognise its significance. Nevertheless, a larger percentage of laboratories demonstrate a growing interest and intent to perform this analysis in the future.
- Reference materials could help in defining quality criteria for the data generated by WWS laboratories, ultimately aiding better decision-making and policy development in the field. A coordinated approach is required to define which reference materials are needed for this purpose.

### Electronic supplementary material

Below is the link to the electronic supplementary material.

Supplementary Material 1

Supplementary Material 2

Supplementary Material 3

Supplementary Material 4

Supplementary Material 5

Supplementary Material 6

Supplementary Material 7

## Data Availability

No datasets were generated or analysed during the current study.

## Abbreviations

AMR: Antimicrobial resistance
COVID-19: Coronavirus disease 19
ddPCR: Digital droplet polymerase chain reaction
dPCR: Digital polymerase chain reaction
EU: European Union
LOD: Limit of detection
LOQ: limit of quantification
NCBI: National Center for Biotechnology Information
PCR: Polymerase chain reaction
PEG: Polyethylene glycol
PT: Proficiency testing
RNA: Ribonucleic acid
RT-PCR: Reverse transcription polymerase chain reaction
SARS-CoV-2: Severe acute respiratory syndrome coronavirus 2
WHO: World Health Organization
WWS: Wastewater surveillance
