# Supplementary material for: EU surveys insights: analytical tools, future directions, and the essential requirement for reference materials in wastewater monitoring of SARS-CoV-2, antimicrobial resistance and beyond
Source: Hum Genomics. 2024 Jun 27;18:72. doi: 10.1186/s40246-024-00641-5 (PMC11210120; doi:10.1186/s40246-024-00641-5)
Supplement: Supplementary file 1 — Supplementary Material 1 [file 40246_2024_641_MOESM1_ESM.pdf]

Reference materials in wastewater surveillance

Fields marked with * are mandatory.

Reference materials in wastewater surveillance

Wastewater surveillance of SARS-CoV2 and other pathogens is based on a wide array of analytical methods and protocols.
We would like to verify whether there is a need to harmonise the various results obtained across the different laboratories operating in this sector.
Reference materials play an essential role in aligning and standardizing results by providing a consistent benchmark for accuracy and precision that can be used to validate and compare measurements across different laboratories, instruments and methods.

By sharing your insights and expertise, you can help identify gaps and opportunities for improving the accuracy and reliability of wastewater surveillance data. Your input will also help guide the development of new reference materials that can improve the comparability and reproducibility of results, ultimately contributing to better decision-making and policy development in the field.

For the compilation of the questionnaire, you will need approximately 5-10 min.

Thank you very much for your time and your precious help.

Do you accept that the scientific information provided in the survey will be used anonymously for internal scoping purposes and eventual future publications?
Personal information will be treated confidentially and will not be disclosed.

- [ ] I accept

1 Participant information

* Participant name

* Email

Telephone number

* Name of institution

* Type of institution

- Accademy
- Industry
- International
- National

* Institution country

- AF - Afghanistan
- AL - Albania
- DZ - Algeria
- AD - Andorra
- AO - Angola
- AG - Antigua and Barbuda
- AR - Argentina
- AM - Armenia
- AU - Australia
- AT - Austria
- AZ - Azerbaijan
- BS - Bahamas
- BH - Bahrain
- BD - Bangladesh
- BB - Barbados
- BY - Belarus
- BE - Belgium
- BZ - Belize
- BJ - Benin
- BT - Bhutan
- BO - Bolivia
- BA - Bosnia and Herzegovina
- BW - Botswana
- BR - Brazil
- BN - Brunei Darussalam
- BG - Bulgaria
- BF - Burkina Faso
- BI - Burundi
- CV - Cabo Verde
- KH - Cambodia
- CM - Cameroon
- CA - Canada

- CF - Central African Republic
- TD - Chad
- CL - Chile
- CN - China
- CO - Colombia
- KM - Comoros
- CG - Congo
- CR - Costa Rica
- CI - Côte D'Ivoire
- HR - Croatia
- CU - Cuba
- CY - Cyprus
- CZ - Czechia
- CD - Democratic Republic of the Congo
- DK - Denmark
- DJ - Djibouti
- DM - Dominica
- DO - Dominican Republic
- EC - Ecuador
- EG - Egypt
- SV - El Salvador
- GQ - Equatorial Guinea
- ER - Eritrea
- EE - Estonia
- SZ - Eswatini
- ET - Ethiopia
- FJ - Fiji
- FI - Finland
- FR - France
- GA - Gabon
- GM - Gambia
- GE - Georgia
- DE - Germany
- GH - Ghana
- GR - Greece
- GD - Grenada
- GT - Guatemala
- GN - Guinea
- GW - Guinea Bissau
- GY - Guyana
- HT - Haiti
- HN - Honduras
- HU - Hungary
- IS - Iceland
- IN - India
- ID - Indonesia
- IR - Iran

- IQ - Iraq
- IE - Ireland
- IL - Israel
- IT - Italy
- JM - Jamaica
- JP - Japan
- JO - Jordan
- KZ - Kazakhstan
- KE - Kenya
- KI - Kiribati
- KW - Kuwait
- KG - Kyrgyzstan
- LA - Laos
- LV - Latvia
- LB - Lebanon
- LS - Lesotho
- LR - Liberia
- LY - Libya
- LI - Liechtenstein
- LT - Lithuania
- LU - Luxembourg
- MG - Madagascar
- MW - Malawi
- MY - Malaysia
- MV - Maldives
- ML - Mali
- MT - Malta
- MH - Marshall Islands
- MR - Mauritania
- MU - Mauritius
- MX - Mexico
- FM - Micronesia
- MC - Monaco
- MN - Mongolia
- ME - Montenegro
- MA - Morocco
- MZ - Mozambique
- MM - Myanmar
- NA - Namibia
- NR - Nauru
- NP - Nepal
- NL - Netherlands
- NZ - New Zealand
- NI - Nicaragua
- NE - Niger
- NG - Nigeria
- KP - North Korea

- MK - North Macedonia
- NO - Norway
- OM - Oman
- PK - Pakistan
- PW - Palau
- PA - Panama
- PG - Papua New Guinea
- PY - Paraguay
- PE - Peru
- PH - Philippines
- PL - Poland
- PT - Portugal
- QA - Qatar
- MD - Republic of Moldova
- RO - Romania
- RU - Russian Federation
- RW - Rwanda
- KN - Saint Kitts and Nevis
- LC - Saint Lucia
- VC - Saint Vincent and the Grenadines
- WS - Samoa
- SM - San Marino
- ST - Sao Tome and Principe
- SA - Saudi Arabia
- SN - Senegal
- RS - Serbia
- SC - Seychelles
- SL - Sierra Leone
- SG - Singapore
- SK - Slovakia
- SI - Slovenia
- SB - Solomon Islands
- SO - Somalia
- ZA - South Africa
- KR - South Korea
- SS - South Sudan
- ES - Spain
- LK - Sri Lanka
- SD - Sudan
- SR - Suriname
- SE - Sweden
- CH - Switzerland
- SY - Syrian Arab Republic
- TJ - Tajikistan
- TZ - Tanzania
- TH - Thailand
- TL - Timor-Leste

- TG - Togo
- TO - Tonga
- TT - Trinidad and Tobago
- TN - Tunisia
- TR - Turkey
- TM - Turkmenistan
- TV - Tuvalu
- UG - Uganda
- UA - Ukraine
- AE - United Arab Emirates
- GB - United Kingdom
- US - United States of America
- UY - Uruguay
- UZ - Uzbekistan
- VU - Vanuatu
- VE - Venezuela
- VN - Viet Nam
- YE - Yemen
- ZM - Zambia
- ZW - Zimbabwe

2 Surveillance

*** 1. Which viruses are you monitoring in your wastewater surveillance programs?**

- Adenovirus
- Chikungunya virus
- Coxsackie virus
- Dengue virus
- Echovirus
- Hepatitis A virus
- Hepatitis E virus
- Influenza virus
- Measles virus
- Monkeypox virus
- Non-polio enteroviruses
- Norovirus
- Poliovirus
- Respiratory Syncytial virus
- Rift Valley Fever virus
- Rotavirus
- SARS-CoV-2
- Yellow Fever virus
- West Nile virus
- None
- Others

* Please specify which other viruse(s) you are monitoring in your wastewater surveillance programs

*** 2. Which bacteria are you monitoring in your wastewater surveillance programs?**

- [ ] *Bordetella pertussis*
- [ ] *Campylobacter spp*
- [ ] *Carbapenem-/colistin-resistant Enterobacterales*
- [ ] *Carbapenem-resistant Acinetobacter baumannii*
- [ ] *Carbapenem-resistant Pseudomonas aeruginosa*
- [ ] *Corynebacterium diphteriae*
- [ ] *Clostridium perfringens*
- [ ] *Clostridioides difficile*
- [ ] *Enteroccus spp*
- [ ] *Haemophilus influenzae*
- [ ] *Klebsiella pneumonia*
- [ ] *Listeria monocytogenes*
- [ ] *Neisseria gonorrhoeae*
- [ ] *Neisseria meningitidis*
- [ ] *Salmonella enterica*
- [ ] *Salmonella typhimurium*
- [ ] *Salmonella Newport*
- [ ] *Shiga toxin/verocytotoxin-producing Escherichia coli*
- [ ] *Shigella*
- [ ] *Streptococcus pneumoniae*
- [ ] *Vibrio cholera*
- [ ] *Total coliform bacteria*
- [ ] None
- [ ] Others

* Please specify which other bacteria you are monitoring in your wastewater surveillance programs

*** 3. Have you ever measured (routinely or not) the presence of antimicrobial resistance (AMR) genes?**

- ○ Yes
- ○ No
- ○ No, but we plan to do it in the future

* Please specify the top three AMR genes you deal with

* **4. Have you ever measured (routinely or not) the presence of bacteria bearing Antimicrobial Resistance (AMR) genes?**

- Yes
- No
- No, but we plan to do it in the future

* Please specify the top three bacterial species you deal with

Which methods do you use?

- [ ] Molecular methods (e.g. PCR, RT-PCR, digital PCR)
- [ ] Sequencing of specific targets
- [ ] Ready-made multi-gene sequencing panels (e.g., Qiagen or Illumina)
- [ ] Metagenomics

5. Do you monitor antimicrobial resistant fungi in your wastewater surveillance?

- Yes
- No
- No, but we plan to do it in the future

Please, specify

6. Do you undertake environmental surveillance?

- Yes
- No

Please specify on which organisms

7. What do you plan to search for in the near future?

8. In your opinion is there a need to improve the accuracy of your results?

- Yes
- No

* 9. Which reference material would you need?

- Whole organism reference materials certified for their identity and concentrations
- Nucleic acid reference materials certified for their sequence and concentrations
- Specific metabolites at certified concentrations
- Others

* Please specify

Any additional comment would like to share with us
