# Supplementary material for: EU surveys insights: analytical tools, future directions, and the essential requirement for reference materials in wastewater monitoring of SARS-CoV-2, antimicrobial resistance and beyond
Source: Hum Genomics. 2024 Jun 27;18:72. doi: 10.1186/s40246-024-00641-5 (PMC11210120; doi:10.1186/s40246-024-00641-5)
Supplement: Supplementary file 2 — Supplementary Material 2 [file 40246_2024_641_MOESM2_ESM.pdf]

Reference materials in wastewater surveillance for SARS-CoV-2

Fields marked with * are mandatory.

Reference materials in wastewater surveillance for SARS-CoV-2

Wastewater surveillance has proven to be a valuable tool in tracking the spread of SARS-CoV-2 and assessing the effectiveness of public health interventions. Your collaboration and knowledge sharing are crucial to further advancements in this field. We kindly request your assistance in sharing your experiences and technical protocols related to SARS-CoV-2 wastewater surveillance.

We are particularly interested in the following areas:

- Sample processing and RNA extraction: Details regarding the most effective techniques for processing wastewater samples to concentrate viral particles and extract viral RNA for subsequent analysis.

- Molecular testing methods: Information on the molecular techniques, such as RT-PCR or other methods, employed for the detection and quantification of SARS-CoV-2 in wastewater samples. This may include specific primers and probes used, as well as any adaptations made for improved sensitivity or efficiency.

- Data analysis and interpretation: Best practices for analysing and interpreting data generated from wastewater surveillance, including strategies for tracking trends, estimating viral load, and correlating results with clinical data.

We look forward to your positive response and the possibility of establishing a valuable exchange of information.

You will need about 10-15 minutes to complete the survey.

Thank you very much for your time and your precious help.

Do you accept that the scientific information provided in the survey will be used anonymously for internal scoping purposes and eventual future publications?
Personal information will be treated confidentially and will not be disclosed.

☐ I accept

1 Participant information

* Participant name

* Email

Telephone number

* Name of institution

* Type of institution

- Academy
- Industry
- International
- National

* Institution country

- AF - Afghanistan
- AL - Albania
- DZ - Algeria
- AD - Andorra
- AO - Angola
- AG - Antigua and Barbuda
- AR - Argentina
- AM - Armenia
- AU - Australia
- AT - Austria
- AZ - Azerbaijan
- BS - Bahamas
- BH - Bahrain
- BD - Bangladesh
- BB - Barbados
- BY - Belarus
- BE - Belgium
- BZ - Belize
- BJ - Benin

- BT - Bhutan
- BO - Bolivia
- BA - Bosnia and Herzegovina
- BW - Botswana
- BR - Brazil
- BN - Brunei Darussalam
- BG - Bulgaria
- BF - Burkina Faso
- BI - Burundi
- CV - Cabo Verde
- KH - Cambodia
- CM - Cameroon
- CA - Canada
- CF - Central African Republic
- TD - Chad
- CL - Chile
- CN - China
- CO - Colombia
- KM - Comoros
- CG - Congo
- CR - Costa Rica
- CI - Côte D'Ivoire
- HR - Croatia
- CU - Cuba
- CY - Cyprus
- CZ - Czechia
- CD - Democratic Republic of the Congo
- DK - Denmark
- DJ - Djibouti
- DM - Dominica
- DO - Dominican Republic
- EC - Ecuador
- EG - Egypt
- SV - El Salvador
- GQ - Equatorial Guinea
- ER - Eritrea
- EE - Estonia
- SZ - Eswatini
- ET - Ethiopia
- FJ - Fiji
- FI - Finland
- FR - France
- GA - Gabon
- GM - Gambia
- GE - Georgia
- DE - Germany
- GH - Ghana

- GR - Greece
- GD - Grenada
- GT - Guatemala
- GN - Guinea
- GW - Guinea Bissau
- GY - Guyana
- HT - Haiti
- HN - Honduras
- HU - Hungary
- IS - Iceland
- IN - India
- ID - Indonesia
- IR - Iran
- IQ - Iraq
- IE - Ireland
- IL - Israel
- IT - Italy
- JM - Jamaica
- JP - Japan
- JO - Jordan
- KZ - Kazakhstan
- KE - Kenya
- KI - Kiribati
- KW - Kuwait
- KG - Kyrgyzstan
- LA - Laos
- LV - Latvia
- LB - Lebanon
- LS - Lesotho
- LR - Liberia
- LY - Libya
- LI - Liechtenstein
- LT - Lithuania
- LU - Luxembourg
- MG - Madagascar
- MW - Malawi
- MY - Malaysia
- MV - Maldives
- ML - Mali
- MT - Malta
- MH - Marshall Islands
- MR - Mauritania
- MU - Mauritius
- MX - Mexico
- FM - Micronesia
- MC - Monaco
- MN - Mongolia

- ME - Montenegro
- MA - Morocco
- MZ - Mozambique
- MM - Myanmar
- NA - Namibia
- NR - Nauru
- NP - Nepal
- NL - Netherlands
- NZ - New Zealand
- NI - Nicaragua
- NE - Niger
- NG - Nigeria
- KP - North Korea
- MK - North Macedonia
- NO - Norway
- OM - Oman
- PK - Pakistan
- PW - Palau
- PA - Panama
- PG - Papua New Guinea
- PY - Paraguay
- PE - Peru
- PH - Philippines
- PL - Poland
- PT - Portugal
- QA - Qatar
- MD - Republic of Moldova
- RO - Romania
- RU - Russian Federation
- RW - Rwanda
- KN - Saint Kitts and Nevis
- LC - Saint Lucia
- VC - Saint Vincent and the Grenadines
- WS - Samoa
- SM - San Marino
- ST - Sao Tome and Principe
- SA - Saudi Arabia
- SN - Senegal
- RS - Serbia
- SC - Seychelles
- SL - Sierra Leone
- SG - Singapore
- SK - Slovakia
- SI - Slovenia
- SB - Solomon Islands
- SO - Somalia
- ZA - South Africa

- KR - South Korea
- SS - South Sudan
- ES - Spain
- LK - Sri Lanka
- SD - Sudan
- SR - Suriname
- SE - Sweden
- CH - Switzerland
- SY - Syrian Arab Republic
- TJ - Tajikistan
- TZ - Tanzania
- TH - Thailand
- TL - Timor-Leste
- TG - Togo
- TO - Tonga
- TT - Trinidad and Tobago
- TN - Tunisia
- TR - Turkey
- TM - Turkmenistan
- TV - Tuvalu
- UG - Uganda
- UA - Ukraine
- AE - United Arab Emirates
- GB - United Kingdom
- US - United States of America
- UY - Uruguay
- UZ - Uzbekistan
- VU - Vanuatu
- VE - Venezuela
- VN - Viet Nam
- YE - Yemen
- ZM - Zambia
- ZW - Zimbabwe

2 SARS-CoV-2 analytical workflow

1. Which method do you use as pre-concentration treatment to inactivate viruses before sample processing?

- [ ] Heat inactivation
- [ ] Chemical inactivation
- [ ] UV irradiation
- [ ] Mechanical disruption
- [ ] Others
- [ ] I do not make any pre-treatment of the sample to inactivate viruses

Please specify

2. What type of viral concentration methods do you use prior to RNA extraction?

- ☐ Filtration
- ☐ Centrifugation
- ☐ Ultracentrifugation
- ☐ Precipitation (e.g. using polyethylene glycol (PEG) or organic solvents
- ☐ Flocculation (e.g. using skim milk)
- ☐ Others

Please specify

3. What RNA extraction method do you use?

- ☐ Commercial kit
- ☐ Ad hoc developed method

4. Which principle is your RNA extraction method based on?

- ☐ (Magnetic) silica bead-based extraction
- ☐ Phenol-chloroform extraction
- ☐ Direct lysis methods
- ☐ Others

5. Please specify your extraction protocol. For instance, specify the name of the commercial kit used or mention the followed international standard (CEN, ISO, DIN,...)

6. Which of the following methods do you use to analyse the SARS-CoV-2 RNA?

- [ ] Real-time Reverse Transcription (RT)-qPCR
- [ ] Droplet or chamber digital (RT-PCR)
- [ ] Sequencing
- [ ] Others

Please specify

7. Which reverse transcriptase (RT)-PCR assays are you using?

- [ ] Commercial assays
- [ ] In-house developed assays
- [ ] WHO recommended assays
- [ ] Others

8. Please specify names and/or references of the assay(s) you are using

9. Which SARS-CoV-2 target gene do you amplify by RT-PCR methods?

- [ ] ORF1ab
- [ ] RdRp
- [ ] N gene
- [ ] E gene
- [ ] S gene
- [ ] Others

Please specify

10. Do you use sequencing methods to analyse the SARS-CoV-2 genome?

- ( ) Yes: targeted approach (amplicon sequencing)
- ( ) Yes: untargeted approach (metagenomics)

- No
- No, but we plan to implement it in the future

3 Quality Control/Quality assurance

11. Which steps have been involved in the validation process?

- The use of a Positive Control
- The use of a Negative Control
- Spike-In Experiments
- Limit of Detection (LoD)/ Limit of Quantification (LoQ) Determination
- Precision and Reproducibility
- Comparison to Established Methods
- Interference Assessment
- Ruggedness/Robustness
- Others

Please specify

12. Which quality control measures are applied?

- Good laboratory practices
- Monitoring and documenting the performance of equipment
- Tracking reagents
- Maintaining a rigorous sample handling and tracking system
- Inhibition control
- Negative extraction control
- Negative environmental control
- Others

Please specify

13. At which step of the complex analytical workflow in wastewater surveillance do you use reference materials?

- Sampling
- Sample pre-treatment

- [ ] Sample concentration
- [ ] Nucleic acid extraction
- [ ] Detection
- [ ] Quantification
- [ ] I do not use reference materials

Why?

14. Do you use reference materials as a surrogate virus in your process control analyses?

- [ ] Yes. Bacteriophage (e.g. MS2 or PhiX174)
- [ ] Yes. Porcine epidemic diarrhoea virus
- [ ] Yes. Bovine coronavirus
- [ ] Yes. Murine hepatitis virus
- [ ] Yes. Murine norovirus
- [ ] Yes. Pepper Mild Mottle virus
- [ ] Others
- [ ] I do not use any reference material

Please specify

15. Which human faecal controls are you using?

- [ ] Pepper Mild Mottle virus
- [ ] Cross-assembly phage (crAssphage)
- [ ] Bacteroides HF183
- [ ] Lachnospiraceae
- [ ] Others
- [ ] I do not use a human faecal control

Please specify the other human faecal controls you use

16. Do you think that the spike-in of surrogate viruses, added in your wastewater sample immediately after sampling, may help to minimize errors and estimate virus recovery from wastewater samples?

- Yes
- No
- Maybe, depending on the spike material

17. If you perform sequencing of SARS-CoV-2 in wastewater samples, how do you control the quality of the sequencing results?

- Computational analysis
- Using standard materials
- I do not control
- Other

18. Have you validated the bioinformatics workflow you use to analyse your data?

- Yes, in house
- Yes, according to National standards
- Yes, according to international standards
- No, but we plan to validate it in the future
- No

19. Are you interested in controlling (and/or benchmarking) bioinformatics software tools that you typically use?

- Yes
- No

Any additional comment would like to share with us

4 GLOSSARY

For terms and definitions, please refer to https://www.iso.org/obp/ui#iso:std:iso:22174:ed-1:v1:en
