# Supplementary material for: EU surveys insights: analytical tools, future directions, and the essential requirement for reference materials in wastewater monitoring of SARS-CoV-2, antimicrobial resistance and beyond
Source: Hum Genomics. 2024 Jun 27;18:72. doi: 10.1186/s40246-024-00641-5 (PMC11210120; doi:10.1186/s40246-024-00641-5)
Supplement: Supplementary file 3 — Supplementary Material 3 [file 40246_2024_641_MOESM3_ESM.pdf]

Additional file 3

Participation to the EU surveys per country in Europe.

Number of invitations sent in Europe per country and number of replies obtained.

European Countries	Invitations	Number of replies for Survey 1	Number of replies for Survey 2
Andorra	1	0	0
Austria	20	4	3
Belgium	21	5	3
Bosnia and Herzegovina	1	1	1
Bulgaria	8	1	0
Croatia	20	3	2
Cyprus	21	2	2
Czechia	10	4	4
Denmark	18	5	1
Estonia	9	1	1
Finland	25	3	1
France	22	8	2
Germany	67	3	5
Greece	23	4	2
Hungary	6	1	1
Ireland	13	1	0
Italy	88	13	11
Latvia	11	1	1
Liechtenstein	0	1	1
Lithuania	4	1	1
Luxembourg	7	2	1
Malta	8	3	2
Netherlands	18	3	1
Norway	5	1	1
Poland	14	1	0
Portugal	37	5	3
Romania	5	2	1
Serbia	0	1	0
Slovakia	20	2	2
Slovenia	9	2	1
Spain	96	9	6
Sweden	22	2	2
Turkey	4	1	1
United Kingdom	38	5	5
**TOTAL**	**671**	**101**	**68**
