# Supplementary figures and images for: EU surveys insights: analytical tools, future directions, and the essential requirement for reference materials in wastewater monitoring of SARS-CoV-2, antimicrobial resistance and beyond
Source: Hum Genomics. 2024 Jun 27;18:72. doi: 10.1186/s40246-024-00641-5 (PMC11210120; doi:10.1186/s40246-024-00641-5)

## Survey participants

Type of institution ● Academy ● Industry ● National

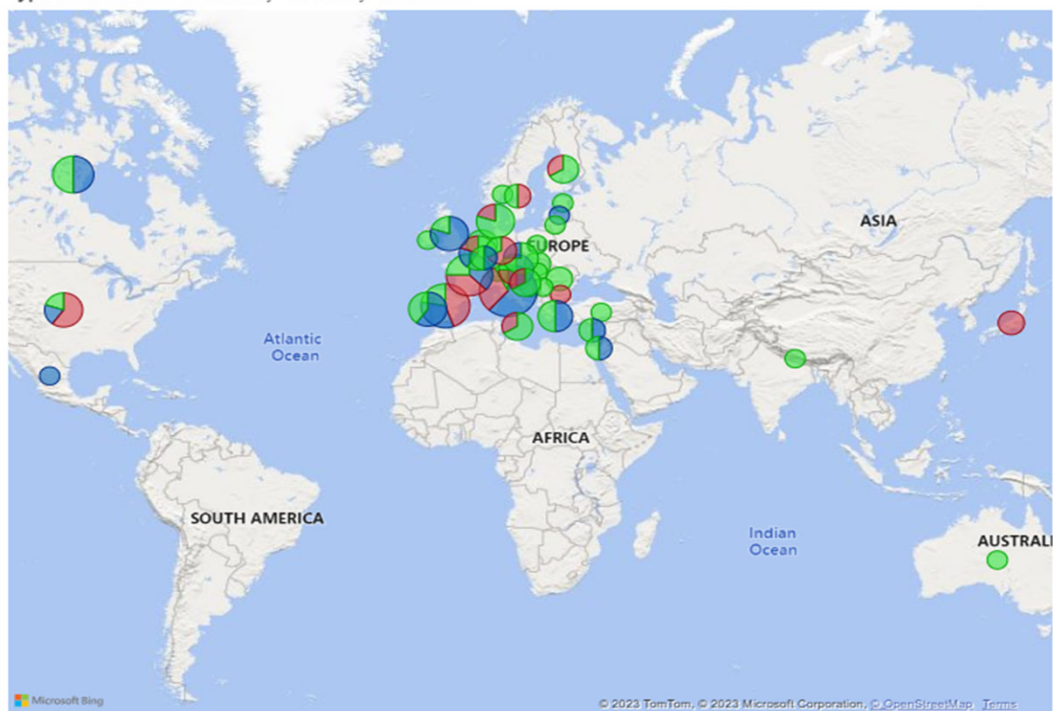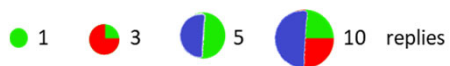

Supplement: Supplementary file 4 — Supplementary Material 4 [file 40246_2024_641_MOESM4_ESM.pdf]

A

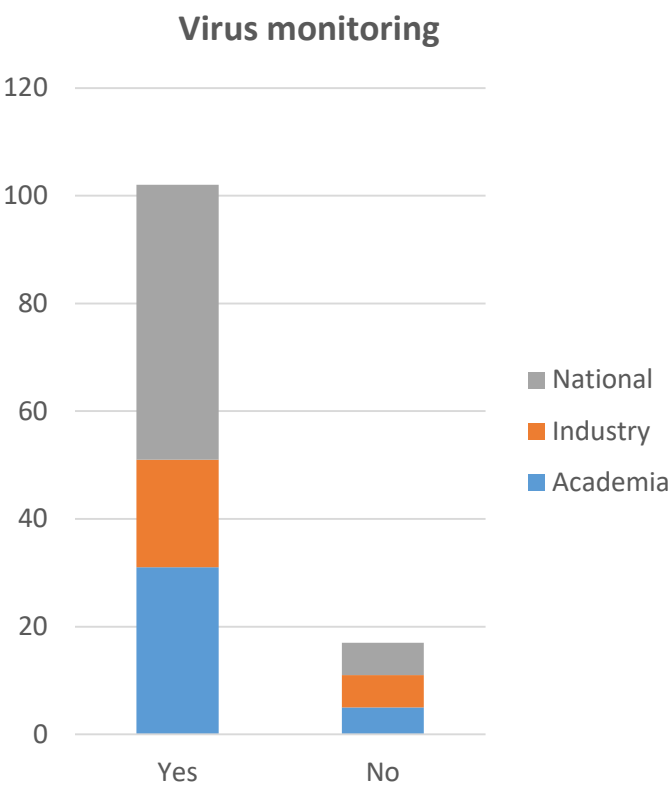

B

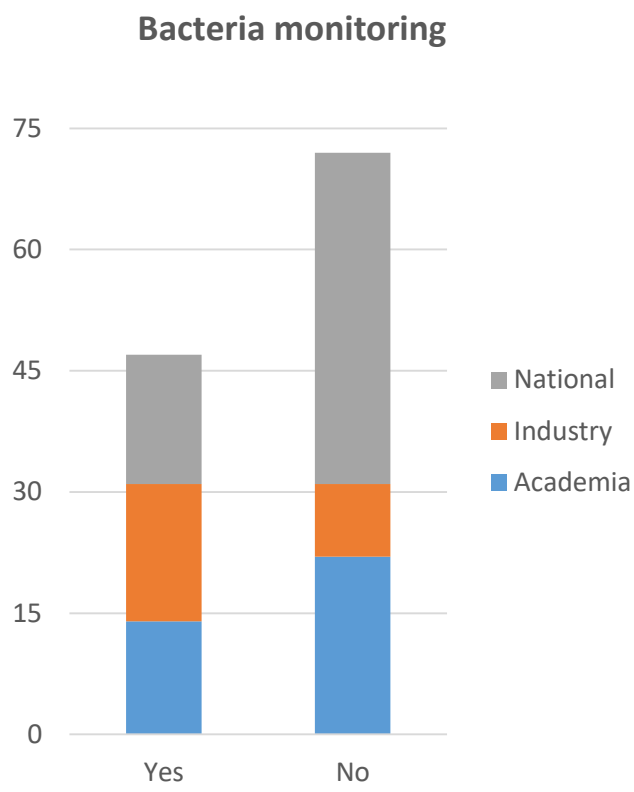

C

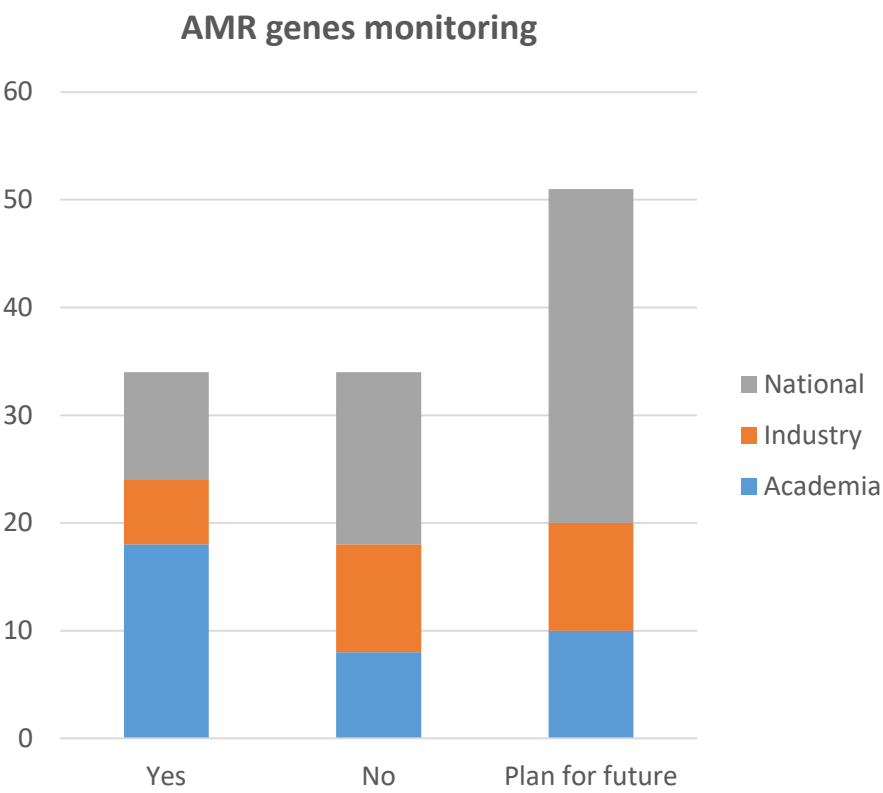

D

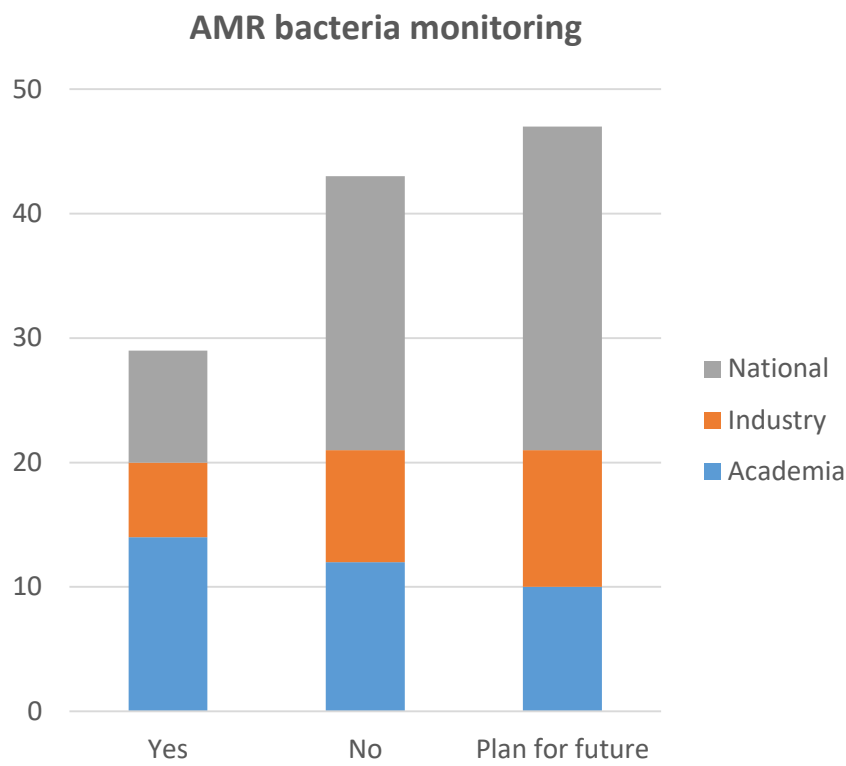

E

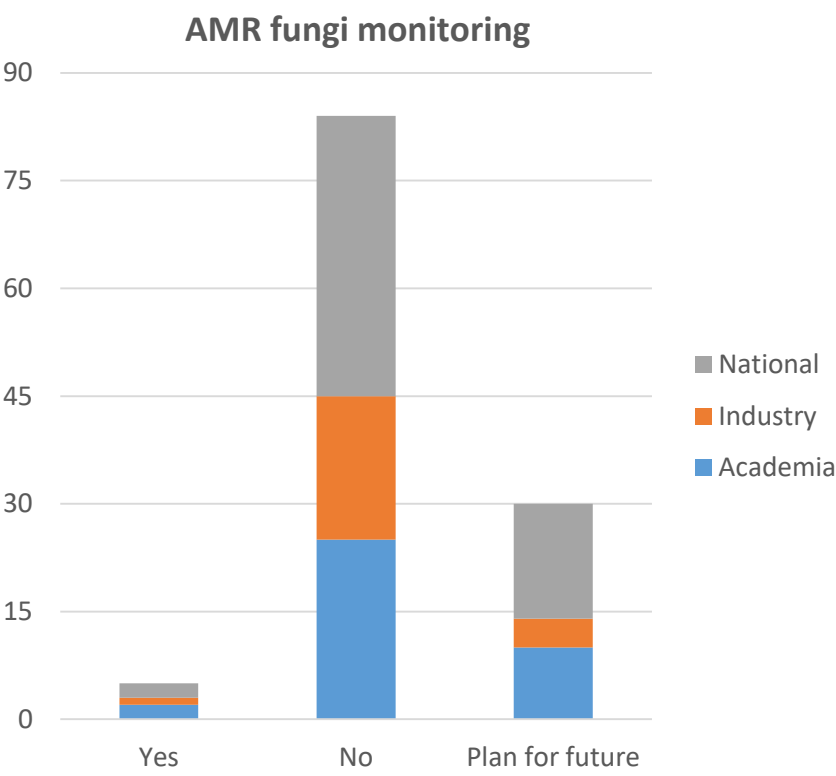

F

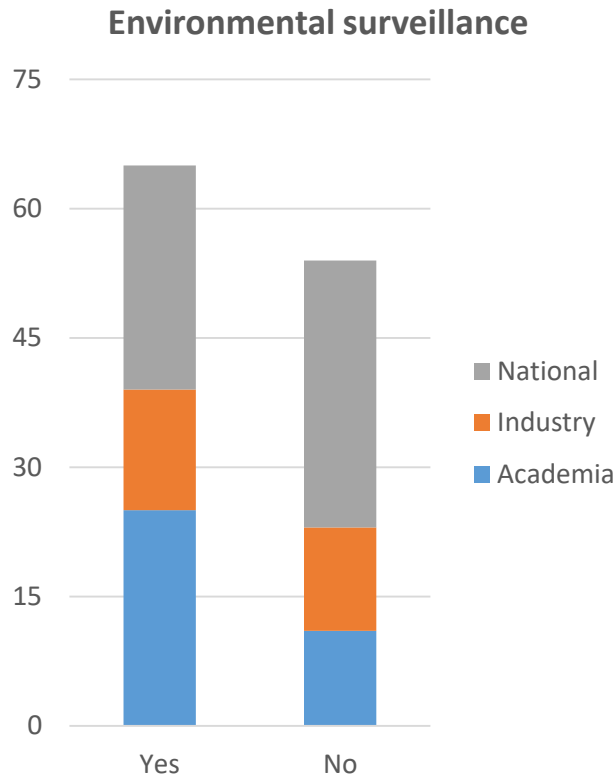

Supplement: Supplementary file 5 — Supplementary Material 5 [file 40246_2024_641_MOESM5_ESM.pdf]

A

## Environmental surveillance

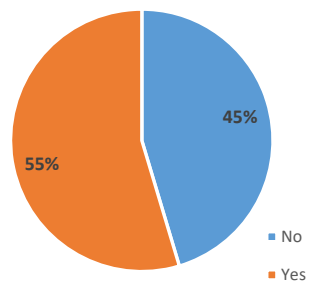

B

## Virus monitored

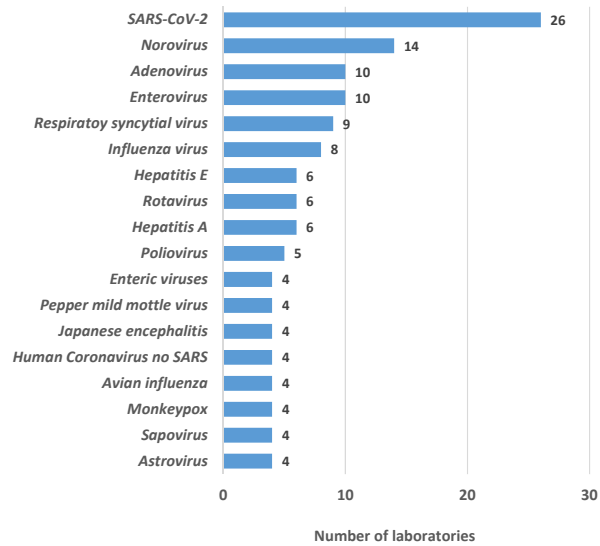

C

## Bacteria monitored

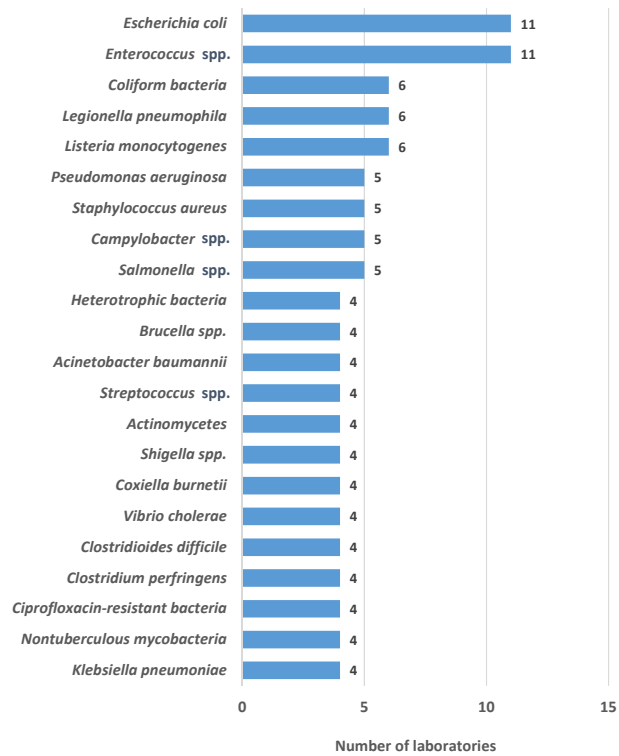

Supplement: Supplementary file 6 — Supplementary Material 6 [file 40246_2024_641_MOESM6_ESM.pdf]

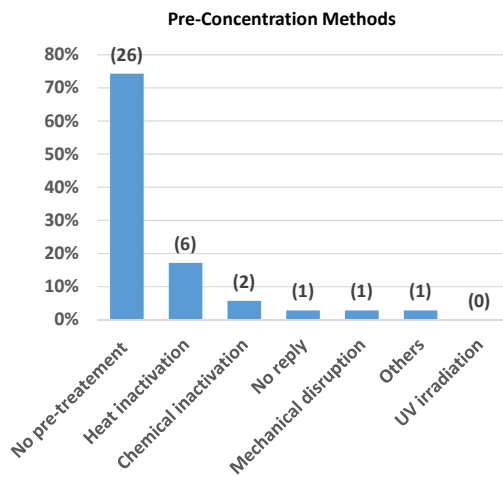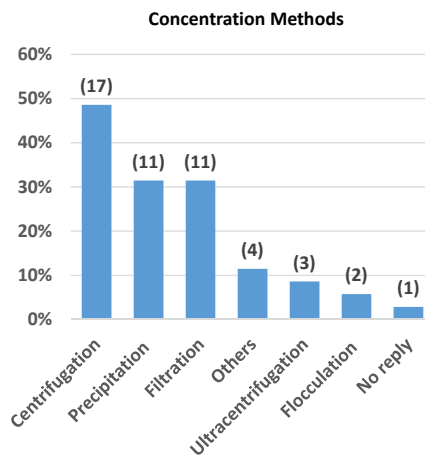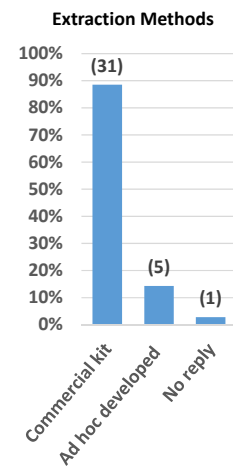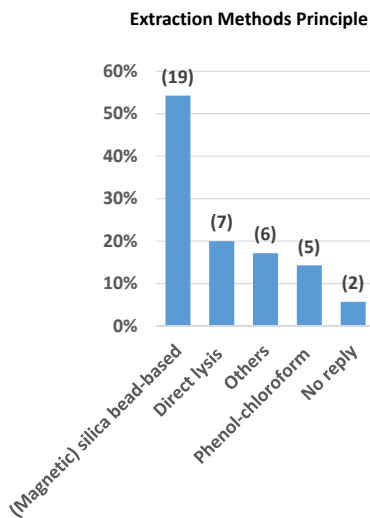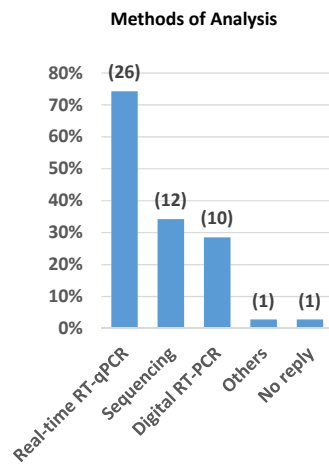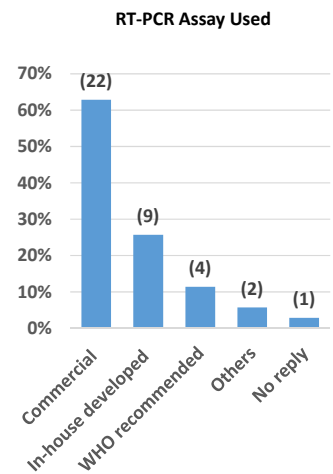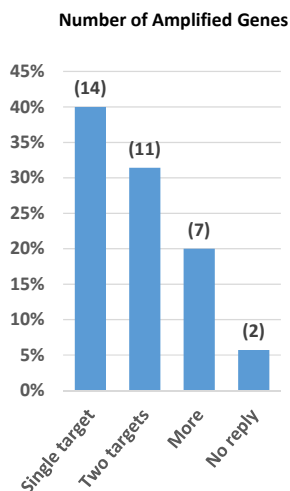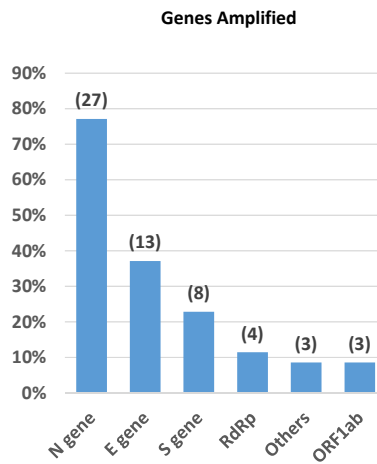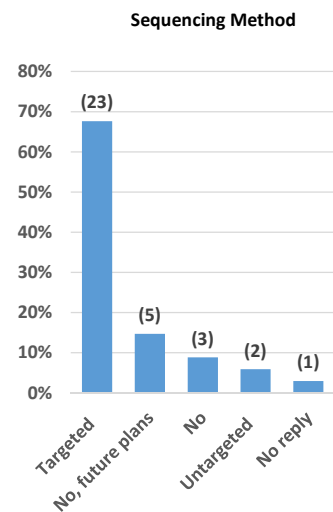

Supplement: Supplementary file 7 — Supplementary Material 7 [file 40246_2024_641_MOESM7_ESM.pdf]
